# The Brain and Its Physiology; a Critical Disquisition on the Methods of Determining the Relations Subsisting between the Structure and Functions of the Encephalon

**Published:** 1846-10

**Authors:** 


### Art. XIII.

*The Brain and its Physiology; a Critical Disquisition on the Methods of determining the Relations subsisting between the Structure and Functions of the Encephalon.* By Daniel Noble, m.r.c.s. — *London,* 1846. Post 8vo, pp. 459.

"The doctrine of the functions of the brain constitutes, most assuredly, a subject of discussion inferior to no other branch of science, either in interest, extent of its objects, or in general importance; and in physiology it must, for many reasons, be esteemed the highest department."

With this prologue to our author's introductory chapter we need scarcely express our hearty concurrence; and notwithstanding that we have on former occasions treated somewhat in full the subject of the physiology of the brain, both in its phrenological aspect and in connexion with the physiology of the nervous system in general, we feel called upon, by the peculiar position of the question at the present time, to enter again into a somewhat detailed examination of it; with the hope of throwing some new light upon it from our own resources, and of being thus able to assist in clearing the path to further discovery. Nothing can be better than the spirit with which Mr. Noble sets out upon his undertaking, as will be evident from the following extract, which follows a notice of the imperfect views current upon the subject amongst physiologists in general:

"And yet there is no good reason why an inquiry into the functions of the brain, and of its various parts, should not be successfully prosecuted; for there is no obscurity in this department of physiological research, which is not common to many others. Cerebral physiology has its own obstacles and its own difficulties, like any other branch of science, requiring certain mental peculiarities and favorable opportunities for their removal and dissipation; but still, under just circumstances, the discovery, not only of the general office of the brain, but of the functions of its particular parts, is an object of pursuit for the philosopher as legitimate, and as likely to reward the labour of investigation, as the solution of any other physiological problem. This attempt will be found useless, or productive of imperfect or contradictory results, only so long as the method pursued is defective or faulty. All sciences, physiological not less than those purely physical, have sustained both error in their development and retardation in their progress, wherever they have been cultivated, without the guidance of sound principles in the investigation. Under such circumstances talent has but led to confusion, and industry has yielded no fruit." (p. 10.)

But what are "the sound principles of investigation" which are to guide us in this difficult department of physiology? Are they those which have been found successful in other branches of physiological inquiry, and which would naturally occur to us as our safest indicators in the exploration of a new and unexplored path? Or are we to discard these tried and faithful guides, and to rely exclusively upon others, which have been long since demonstrated to be insufficient to conduct us to a valid conclusion, in almost every other portion of the inquiry into the functions of the human structure? At this starting point, we regret to find ourselves so far from agreeing with Mr. Noble, that our further paths must be widely divergent. For the main object of his work is to prove, that the physiology of the brain of man must be first evolved by the comparison of the relative development of its different parts, in different individuals of the human race, with the proportionate manifestation of the various powers, propensities, &c., in the characters of these individuals respectively; and that the validity of the inferences derived from comparative anatomy must be tested by their coincidence with those thus deduced from observation on man. We, on the other hand, are prepared to maintain that comparative anatomy is here, as elsewhere, our most trustworthy guide; and that the validity of any inferences drawn from observation of the coincidence between the development and the functional manifestations of the various parts of the human encephalon, must be tested by their conformity with its obvious and decided indications.

We must do Mr. Noble the justice to say that he makes out what will doubtless appear to a large proportion of his readers a most excellent case. He hits away, right and left, at comparative anatomists and vivisectors, and at physiologists who pin their faith upon them; and demolishes all their assertions and arguments with such cleverness, and so completely to his own satisfaction, that none but those who have previously made themselves well acquainted with the subject are likely to do otherwise than accord with him. His confidence in the fundamental correctness of the method of Gall is so great, that he unhesitatingly undertakes to defend it against all comers; his admission of possible improvements in it being so limited as not at all to affect the ardour in which he upholds it, as affording the only sound exposition of the functions of the encephalon.

But he doos not give us any sufficient explanation why, in this particular case, we are to abandon that philosophical method of studying physiology upon the basis of comparative anatomy; the advantages of which are every day becoming more apparent. His arguments on this point may be reduced to these two:—the supposed difficulty of determining the real analogies of the several parts of the nervous system, in animals constructed upon different types;—and the want of materials for a reasonable psychology of the lower tribes of animals. Now in regard to the first point, we do not hesitate to say that the difficulty is more apparent than real. Mr. Noble has made the most of it, by pointing out the discordance on this subject in the writings of comparative anatomists; but this discordance was much greater in the infancy of the science than it is now, and it is gradually giving way to a very satisfactory harmony of opinion, as the principles upon which the investigation must be conducted are being gradually evolved. Such discrepancies are common to the early stage of all sciences. It would be equally to the purpose to quote the early mistakes of Gall, the unadmitted novelties of Vimont, the host of new organs established (to their own satisfaction) by the mesmero-phrenologists, but not admitted by their more conservative leaders, or the discrepancies between the London, Edinburgh, and Parisian professors of the science, in proof of the impossibility of drawing any safe or valid deductions from the method of observation which they advocate. And concerning the second objection we have to remark that, with regard to the broad distinctions between reflex (or excito-motor), instinctive (or sensori-motor), and intelligent (or volitional) actions—which distinctions, as we shall presently show, are of fundamental importance in the inquiry,—we think that ample materials exist for determining them, if they are rightly employed. In fact, in the prosecution of both these inquiries the great evil seems to us to have been, that we have taken man as the type, and have endeavoured to refer everything to our own standard; whereas, if we had investigated the structure of the nervous system in the different classes of animals, and studied their actions, *from below upwards*, we are confident that much greater progress would now have been made.

In no department of physiology, as it seems to us, is the necessity of a continual appeal to comparative anatomy more obvious, than in the inquiry into the functions of the nervous centres. There is none in which we can more clearly trace the withdrawal of one organ after another as we descend the scale, leaving us a remainder whose operations we can study without the disturbing influences of vivisection. There is none in which we can more readily trace (if we only know how to look for it) the distinction between what is superadded and what is essential—what is apparent and what is real. There is none, then, in which we can so completely make use of the lower animals, in Cuvier's happy expression, as so many experiments ready prepared for us by nature. And there is none in which the neglect of this guide is more likely to lead us astray. We say this with no hostility towards the phrenological school; for, as will hereafter appear, we think ourselves able to reconcile the leading dogmata of both systems, in a degree which we have not until recently thought possible; and we have already, on several occasions, expressed our leanings in its favour. But we are desirous that all inductions respecting the physiology of the Encephalon should have the soundest possible basis; and we are convinced that the best method of ascertaining what are the functions of its several parts, is to study those conditions in which their development presents the most striking contrasts, before proceeding to the comparison of them in different individuals of the human race, amongst whom there is not by any means a like diversity. Thus we are surely better able to estimate the the functions of the cerebrum as a whole, and thus to lay the foundation for the inquiry into the distinct operations of its several lobes and minor subdivisions, if we first determine what it does *not* do, by tracing it through the various degrees of degradation which it exhibits as we descend the vertebrated series, until it entirely disappears in the lowest; and by observing the actions of animals, which are destitute of the remotest analogue of it—than by any observation of even the extremest diversities, which are presented in its development in the human race. From not attaching what appears to us sufficient importance to this kind of investigation, Mr. Noble devotes his almost exclusive attention to the functions of the cerebrum and cerebellum; his allusions to the existence of any other ganglionic centres not being by any means pointed, except where he combats the opinions of Drs. Carus and Carpenter upon the functions of the tubercula quadrigemina. Consequently, though professing to be a treatise on the brain, his book is really devoted almost exclusively to an exposition of the functions of the cerebrum and cerebellum alone. Nevertheless, from some hints let fall here and there, we gather that Mr. Noble is inclined to admit that the primary seat of sensation is not in the gray surface of the hemispheres, but in the masses of ganglionic matter at the base of the brain. Now, as we shall presently endeavour to show, the existence of a series of ganglia in immediate connexion with the organs of sense, is a neurological fact of far greater generality than the existence of a cerebrum, and is, therefore, of more fundamental importance; and we by no means despair, therefore, of convincing Mr. Noble and those phrenologists who like him are real truth-seekers, of the justice of our views regarding the primary functional value to be attached to these bodies.

Our present object is, rather, to indicate the mode in which the physiology of the encephalon ought, in our opinion, to be studied, than to enter upon a detailed criticism of Mr. Noble's very clever treatise; since there are many circumstances which lead us to refrain from the latter course, whilst we think we can do some service to science by adopting the former. But in justiçe to Mr. Noble we feel it right to state, that we know of no treatise which is calculated to convey to an intelligent and discriminative reader a better idea of phrenology; as he is alike free from the shallow dogmatisms of some authors, and the bold and ignorant quackeries which are so repulsive in others; and, if the correctness of his fundamental principles be conceded, makes a legitimate and philosophical use of observation for the establishment of the truths of which he is in search. Such might be expected from the author of "True and False Phrenology," and of other valued contributions to medical science.

There are some other points, however, which we must discuss with him, before we can altogether dismiss him from our critical bar; deeming him in special need of wholesome correction therefrom. Such, for example, we find in the mode in which he has handled the next object of inquiry,—the use of vivisections, in determining the functions of the nervous system; in commenting upon which Mr. Noble has, we think, greatly underrated the amount of information to be derived from experiments. We cannot call to mind a single point of fundamental importance, that has been established in this department of physiology without such aid. Where would have been our knowledge of the general seats of sensation and of the motor power, if the severance of the nerve-trunks had not revealed to us, that connexion through their means with the nervous centres is essential to the sensibility of every part of the body, and to the subjection of its contractility to the will? Where would have been our knowledge of the relative functions of the fibrous and vesicular (or white and gray) portions of the nervous substance, if experimental research had not informed us, that the former possesses no power save that of conduction, and that wherever the latter presents itself there is an independent power of origination? Where would have been our acquaintance with the relative functions of the anterior and posterior roots of the spinal nerves, had it not been for the vivisections of Sir C. Bell, Magendie, Müller, and others? Mr. Noble thinks that experiments can only be valuable, where there has been some antecedent knowledge gained by analytical comparison of function and structure; but all that anatomical inquiry impressed upon the mind of Sir C. Bell, before commencing his experiments, was that these roots must have different functions; and it is notorious that he had formed an erroneous idea of that difference, and that it rested with experiment to set him right. Where, again, would have been our knowledge of the different functions of the cranial nerves—of the purely motor character of some, of the mixed endowments of others—of the absence of common sensibility in the nerves of special sensation, and of many other similar particulars,—but for experiment? True it is, that minute anatomical inquiry might have rendered many of these inferences probable; but it needed the confirmation of experiment to establish them; and we cannot call to mind a single instance in which there is a discrepancy between the results of these two modes of inquiry. We might advert to the purely motor character of the facial nerve, the purely sensory endowments of the first and second branches of the fifth pair, the mixed endowments of its third division, the almost purely afferent character of the glosso-pharyngeal, the relative afferent and motor powers of the superior and recurrent laryngeal nerves, the specially motor endowments of the pharyngeal branches of the par vagum, and many other such determinations, as resting with equal security upon both these foundations. Where, again, would have been our whole knowledge of the independent endowments of the spinal cord, and of its functional analogy with the ganglionic chain of the articulata, but for vivisections? And yet Mr. Noble would have his readers to believe that little reliance is to be placed upon them; and why? Because the inferences which have been drawn from them do not always accord; the true interpretation of the results not having always been given at first. We wonder that his penetrating mind should have overlooked the fact, that where the first inferences have been demonstrated to be fallacious, that demonstration has been afforded by *additional experiments*.

Mr. Noble quotes, among other illustrations of the uncertainty of such results, the well-known error into which Sir C. Bell was at first led, in regard to the supposed motor functions of the infra-orbital nerve. The ass, in which this nerve had been divided on both sides, stooped down to the oats that were placed before him on the ground; and though the oats touched his lip, he made no effort to pick them up: whence Sir C. Bell inferred that the power of moving the lip had been destroyed by the operation. Now we could not have a better illustration of the necessity of cautiously separating the phenomena, from the inferences founded upon them by the experimenter. Every subsequent vivisector has confirmed this fact; the result of the experiment is invariable. But the inference was wrong, and is now universally admitted to have been so; for it is not the motor power which has been destroyed, but only the sensory; and the animal, not being able to feel the oats, makes no effort for their prehension. And how was this correction supplied? By the additional experiments of Magendie and Mayo; who applied other tests, and proved the persistence of the motor power whilst the sensory was abolished. What ground, then, does this, or any similar fact in the history of neurology, afford for cavilling at the results of experimental research, as uncertain and fallacious? Does Mr. Noble feel less confidence in the now universal accordance of physiologists in Sir C. Bell's determination of the relative functions of the anterior and posterior roots of the spinal nerves, because his inferences were at first incorrect, or because, even when better established, they were for a long time disputed among physiologists; or because whilst *these* are generally received, others of his doctrines (such as his idea of the relative functions of the anterior and posterior columns, or the special respiratory system of nerves) have not gained equal currency, or are altogether rejected? In no science do we find that the whole truth is disclosed and established at once, even when the most sagacious investigators are in pursuit of it; and, considering the peculiar difficulties which attend all physiological researches, and especially those in which the nervous system is involved, we should rather feel surprised that so much has been done in the short time that has elapsed since the path of inquiry was first opened up, than sceptical as to the power of experiment and observation, judiciously conducted, to establish much of what we seek to know.

We would not be thought, however, to be over-sanguine as to the results we expect from vivisections practised upon the encephalon. We have never been disciples of the school of Magendie; and we should shrink from being supposed to sanction the reckless barbarities of those, who have adopted him as their master and guide. We do not disguise from ourselves that any disturbance of the proper balance of pressure in the contents of the cranium is likely to affect the condition of every part; and that it is difficult, if not impossible, to effect the entire severance of any part from its connexions with the rest, without thereby disturbing the normal functions of those which remain. We cannot, therefore, feel the same confidence in the results of vivisections practised upon the encephalon, taken alone, as we should upon those of the division or irritation of a nerve. But if the results of experiments tally sufficiently well with the inferences which seem to flow legitimately from comparative anatomy,—if we find an animal, for instance, from which a certain organ has been removed, to be reduced to the same level with one which is naturally destitute of it, allowance being made for the disturbance produced by the immediate shock of the operation,—or if we find that the removal of another organ uniformly destroys a power, which seems to be possessed by different animals in a degree that accords with the development of that organ, — we unhesitatingly say that it must be very cogent and well-founded evidence, which should lead us to put aside such a determination. The value which we attach to vivisections upon the *brain*, may not, after all, be very different from that set upon them by Mr. Noble; since we have confidence in their results, only when they have been made in harmony with the structural divisions pointed out by nature, and under the guidance of a reasonable idea of the functions of the parts experimented on. But *we* should found these preliminary ideas, both as to structure and function, upon the wide and (in our opinion) secure basis of comparative anatomy; which Mr. Noble would attempt to exclude, as affording no sufficiently positive data. Whether it does so, or not, time and good sense must decide.

Another point on which Mr. Noble has fallen into mistake, through the want of a sufficiently extensive acquaintance with comparative anatomy, and on which we believe that he participates in a common error (which therefore calls the more loudly for correction), is the following. In replying to objections raised against the phrenological system, on the ground that it is unsustained by any direct anatomical proof, he says (p. 237), "Objections such as these can only arise from singular thoughtlessness, or from utter ignorance of nature's plan in the development of the nervous system, which, *as a rule, exhibits no mechanical divisions coincidentally with distinctness in function.*" [The italics are Mr. Noble's.] We do not overlook the connexion in which this sentence occurs; from which it may be inferred that Mr. Noble had the cerebrum of vertebrated animals alone in his mind, and merely intended to account for its not being mapped out like a phrenological bust. But the prominence given to the latter part of it is certainly calculated to mislead; for the applicability of the statement is limited to vertebrated animals, the *general* fact being very different. For no one can take even a cursory view of the development of the nervous system throughout the entire animal series, of which the invertebrated classes form *by far the largest part*, without perceiving the almost invariable rule in those classes to be, that mechanical divisions of the nervous centres *do* coincide with distinctness of function. We have ganglia for respiration, ganglia for mastication and deglutition, ganglia for locomotion, ganglia for special sensation, and ganglia for various actions peculiar to the species (as those connected with the suckers of the cuttle-fish), all of them distinct from each other, and easily recognized by their connexions with the organs specially destined to these functions respectively. In the higher forms of some of the most elevated classes, both of the articulated and molluscous series, we do trace that tendency to fusion and concentration, which is characteristic of the vertebrated sub-kingdom; but even in them the original mechanical distinctness of the ganglia that are functionally different, may be traced by sufficient attention to the history of their development. Thus the large ganglionic masses which occupy the thorax in many insects, and which are almost the only nervous centres in their trunks, are clearly formed by the fusion of some of the ganglia that were distinct in the larva, and by the increased development of the compound centres thus formed, to the exclusion of others which diminish and even disappear altogether. Now in the vertebrated series, which, in any general survey of this kind, must be considered as only *one* out of *four* distinct types, we find a still greater tendency to fusion or union of different ganglionic centres; but the purposes of this are evident,—to place them all under the protection of the cranio-spinal envelope, and to bring them into closer relation, in order to secure greater consentaneousness of action. But does not every physiologist know that in spite of external indications, real mechanical divisions still exist? Are not the segments of the spinal cord as independent of each other, in regard to their power of ministering to the reflex actions of the nerves proceeding from them, as are the distinct ganglia of the ventral cord of the articulata? Is the tract of vesicular matter, which forms the centre of the respiratory nerves, less a respiratory ganglion, because it is imbedded in the medulla oblongata? Is the gray nucleus, into which the root of the auditory nerve may be traced, less a mechanically distinct auditory ganglion because it happens to be imbedded amongst strands of white fibres connecting the organs above and below it?

But even were we not allowed to push our anatomical inquiries so far, as to determine by their means that what bears the external aspect of a single ganglionic mass is in reality formed by the fusion of several, the argument is all the stronger for the functional distinctness of those parts, which *do* remain mechanically distinct, even in the vertebrated series; and we cannot but think that we shall be better guided in our inquiries, by following out the clue afforded us by the divisions which nature has established, and of which she still leaves us the external indications, than by setting out with the establishment of new and arbitrary divisions of our own. Surely the condition of the sympathetic system could never have occurred to Mr. Noble's mind; presenting to us, in the highest vertebrated animals, precisely what we have represented as the *most general* condition of the nervous centres, viz. functional independence, combined with mechanical distinctness. It is not strange that, in the infancy of comparative neurology, the sympathetic system of vertebrata should have been compared with the entire nervous system of invertebrated animals, consisting (as both do) of ganglia scattered as it were through the entire body. Nor is it difficult, on the other hand, to see why the sympathetic system should retain that condition, even in the highest vertebrata, since its offices are not of such predominant importance as to require the protection of the cranio-vertebral column; nor is there such need of consentaneousness in the actions of its different parts, as to require that close connexion between them, which we see so remarkably effected by the union of the centres altogether making up the cerebro-spinal apparatus.

We have made these remarks at this stage of our inquiry because, as we shall next endeavour to show, there is a much greater amount of mechanical distinctness (we use Mr. Noble's phrase in default of a better) in the encephalic centres of man and the higher vertebrata, than is generally supposed. We cannot have a better opportunity of demonstrating the importance of a due acquaintance with comparative anatomy to him who attempts to specialize the functions of the different parts of the human encephalon, than by contrasting the ideas we are led to form of the fundamental divisions of this complex organ, by the study of its anatomy in man alone, with those which we gain from a more extended survey of its development, in the series of animals constructed upon the same general model. The attention of every mere human anatomist (and we are not aware of any phrenologist who forms an exception to this statement) has been fixed almost exclusively upon the two organs, distinguished as the *cerebrum* and the *cerebellum,* as forming, with the medulla oblongata, the entire encephalon. It is not surprising that it should be so, when the preponderating bulk of these organs is considered, and when it is further borne in mind, that these are the only masses which *appear* to have any distinctness—the only ones, indeed, which can be proved to possess any speciality, except by the very closest scrutiny, guided by the light thrown on the search by comparative anatomy. But, go to the opposite extremity of the vertebrated series, and examine the encephalon of a fish: what is there to be found? A series of at least *four* distinct ganglionic masses, arranged in a line continuous with the spinal cord, three of them in pairs, the fourth and hindmost single. The analogues of these in the human encephalon cannot be determined without the most careful scrutiny, both into the relative connexions of the parts, and into the relative degrees of development, and the changes of form and position, which they present in the several intermediate groups, which connect the lowest with the highest of the vertebrated series. It cannot be said that this study, the importance of which has only been recognized of late years, has yet been prosecuted in such a manner as to place every analogy in its clearest and most definite form; but the following points may, we think, be regarded as established by the general consent of those most competent to form a judgment on the subject.

1. The most anterior of the ganglionic masses in the encephalon of the fish, on either side of the median line, is the *olfactory* ganglion, being the centre in which the olfactory nerve terminates. Its connexions are with the most anterior extremity of the medulla oblongata, no evident communication of a direct character being traceable between the olfactive ganglion and the masses which lie immediately behind it. Not unfrequently we find that the olfactive ganglia are separated by a considerable interval from the remainder of the encephalon, being carried forwards in order to be in more immediate apposition with the organ of smell; and being connected with the medulla oblongata by slender peduncles. Hence they have been frequently overlooked, and the peduncles have been mistaken for nerve-trunks, proceeding from the second pair of ganglionic masses. This mistake has been common in human anatomy also; the real ganglionic character of the bulbous expansions, which lie on the cribriform plate of the ethmoid bone, having been overlooked, and the peduncles or commissures, which connect them with the other parts of the encephalon, having been consequently ranked as the first pair of nerves. There can now, however, be no doubt or mistake about the matter, since the presence of vesicular matter in these bulbous expansions, even in man, clearly demonstrates their ganglionic character; and in many of the lower mammalia, in which they are relatively of much greater size, they include a distinct ventricle.

2. The second pair of ganglionic masses in the brain of the fish is now regarded, we believe, by all the most eminent anatomists (whatever may have been their former differences of opinion), as the sole representative of the *cerebral hemispheres* of the higher vertebrata. But even this it is not *in toto*, for the fact appears to be, that only the external lamina of each ganglion, which in most fishes is very thin, can be at all compared with the cerebral hemisphere, the interior mass being rather to be considered, from its aspect and connexions, in the light of a corpus striatum. And we shall presently find reason to believe, that this rudiment of a cerebrum does not represent the entire organ, any more than the early embryo can be regarded as a complete *homunculus*, but that it must be considered as the first indication of the presence of the *anterior lobe* alone.

3. The third pair of encephalic masses has received the name of the *optic lobes*, being evidently the ganglionic centres of the optic nerves. On closely scrutinizing their structure and connexions, it appears that they contain the analogues of the corpora quadrigemina and thalami optici of higher animals, the roots of the optic nerves being especially connected with the former, which must be regarded as the representatives of the optic ganglia of the invertebrata.*

* The first attempt at a philosophical comparison between the encephalon of vertebrated and invertebrated animals, we believe to have been made by Mr. Owen in regard to the brain of the cephalopoda; in the illustrated catalogue of the Museum of the College of Surgeons, vol. i, 1835; and in his article "Cephalopoda," in the Cyclopædia of Anatomy and Physiology, vol. i, 1836. In 1838, Mr. Newport, in his prize essay upon the Athalia centifoliæ, stated it as his opinion, that the cephalic ganglia of insects are analogous to the tubercula quadrigemina of the brain of vertebrata; a statement fundamentally true, but requiring (as we shall presently see) some qualification, since the cephalic ganglia are made up of at least four coalesced pairs, of which the optic ganglia form only one.

4. Lastly, we find a ganglionic mass placed on the median line, over that space left by the divergence of the fibrous strands of the medulla oblongata, which constitutes the fourth ventricle. This mass is evidently the analogue of the *cerebellum.* It sometimes presents the rudiments of lateral appendages; and, as we shall hereafter see, it varies more in its relative dimensions in different species of fish, than does any other of the encephalic ganglia.

Now, that the same distinction of parts exists in the human encephalon is clearly proved by the fact, that all these—with the exception of the olfactive ganglion, which always exists in an almost rudimentary state—are to be most clearly made out in the early embryo. The whole condition of the encephalon (making allowance for the difference between an embryonic and an adult structure) is most remarkably accordant with that of the encephalon of the fish, for it is composed of a series of rounded masses, placed in a curved line, which is continuous with that of the spinal cord, and none of them has any evident predominance over the rest. Of the most anterior of these, the superficial portion is the rudiment of the cerebral hemispheres, whilst the deeper-seated part of the mass is evidently the representative of the corpora striata. In place of the single pair of ganglia behind these, which usually combines in fishes the analogues of the corpora quadrigemina and thalami optici, we have first a vesicle within which the thalami optici and the third ventricle are formed, and then another, which gives origin to the corpora quadrigemina. The mechanical division is here more complete, therefore, than that which prevails in most fully developed fishes, though there are some, as the lamprey, which retain more of the embryonic type, and present, even in their adult condition, two distinct lobes for the thalami optici and corpora quadrigemina. Behind the vesicle from which the latter are formed, in the human embryo, is a large vesicle, within which the cerebellum is developed.

We have, then, in fishes, and in the early human embryo, this remarkable condition of the encephalic centre,—that its great mass is evidently composed of a series of distinct ganglionic centres, of which the portion representing the cerebral hemispheres is usually the smallest. We have—1st, the olfactory ganglia; 2d, the corpora striata, covered by a thin lamina, which is the rudiment of the cerebral hemispheres; 3d, the thalami optici, inclosing the third ventricle; 4th, the corpora quadrigemina; and 5th, the cerebellum. *There is no more general fact in the whole range of comparative anatomy,* than that the encephalon of vertebrata is composed of these elements at the commencement of its development; and however much their relative size and importance may be altered in the progress of their subsequent evolution, their original distinctness remains unaltered. Even in the adult human brain this distinctness may be recognized by careful dissection; for, if we take the medulla oblongata as our guide, and trace its connexions with the several ganglionic masses at its summit, we find it sending distinct fasciculi of fibres into the cerebellum, the corpora quadrigemina, the thalami optici, the corpora striata, and the peduncles of the olfactory ganglia. The cerebellum is not more "mechanically" distinct from the cerebrum than are the corpora quadrigemina, the thalami optici, and the corpora striata from the true cerebral hemispheres. Before attempting, then, to determine the functions of the compound mass, which, taken as a whole, we are accustomed to designate as the encephalon, it appears to us essential that we should distinguish the relative offices of the true cerebral hemispheres, and of the chain of ganglia which they inclose and overtop. We shall otherwise be in continual danger of attributing to one what are really the attributes of the other; and of reasoning upon that as a whole, which is really made up of a series of parts, alike mechanically and functionally distinct. Common sense, we apprehend, would dictate to us, that in the study of a complex piece of machinery, we should first make ourselves acquainted with the actions of the parts, which are sufficiently isolated to enable us to observe their operations independently of those of the remainder, and to put aside from the sum total all that can be fairly attributed to these, before endeavouring to analyse the operations of the portion that most defies our scrutiny. It is absurd to suppose that there is any such complete alteration in man of the relative functions of the several parts, as shall do away with the necessity for regarding them as distinct; when the whole history of the development of the human encephalon indicates its precise accordance with the general type of structure which prevails in the nervous centres of vertebrata, and when nothing is needed but careful scrutiny, to discover the same distinctness in these parts in the *complete* brain, as in the *embryonic.*

We must not, however, quit our account of the fish's encephalon without remarking that, although the ganglionic masses we have enumerated are those most constantly distinguishable, there are others which are probably always present, though usually *fused* into the medulla oblongata,—such as the ganglia of the auditory nerve and of the par vagum, which are "mechanically" distinct in some species, especially those of the carp kind,—whilst there are others, again, which are evidently called into existence to supply some special requirement. Under this last category we may rank the electrical lobe, an additional ganglionic mass peculiar to the gymnotus, from which proceed the greater part of the nerves that supply the electrical organs; a median ganglion, occupying nearly the same situation, but having a function (as known by its connexions) entirely different, in the remora, or sucking-fish, the remarkable suctorial disk on the head of which is supplied from it; and a series of ganglionic enlargements in the medulla oblongata of the trigla, or gurnard, which supplies nerves to the peculiar digitiform prolongations of its pectoral fins. We have noticed these facts to show that the connexion between "mechanical distinctness" and diversity of function is still a very obvious one, notwithstanding the tendency to the fusion of different parts of the nervous centres, which is generally so remarkable in vertebrata.

If, then, we consider the encephalon of vertebrata under its *most general* aspect—that, namely, which all vertebrated animals present at an early period of their development—we find it to consist essentially of the following parts. In the first place, of the medulla oblongata, or cranial prolongation of the spinal cord, which usually includes the respiratory, auditory, and gustatory ganglia, and of which the several strands have distinct connexions with the other ganglionic masses in the encephalon. Secondly, of olfactive and optic ganglia, whose functions are known by their immediate connexion with the nerves of those senses respectively. Thirdly, of masses representing the thalami optici and corpora striata of the human brain, which are here seen to have an existence quite independent of that of the cerebrum, instead of being mere appendages of it. Fourthly, of the cerebellum. And fifthly, of a rudiment of the cerebral hemispheres. When it is borne in mind, that of the second pair of ganglionic masses in the fish's encephalon (which is very frequently smaller than the optic lobes, and seldom much larger) only the external layer or crust is the representative of the real cerebrum, we at once see what a very subordinate part it must perform in the sum-total of the operations of the nervous centres of these animals. The main instruments of these operations must evidently be the *chain of ganglia connected with the sensory organs,* in which, for the reasons we shall presently mention, we include the thalami optici and the corpora striata; and the encephalon is found to be more and more exclusively made up of them, the lower we descend in the scale, until the rudiments of the cerebral hemispheres disappear altogether.

We shall next briefly inquire into the analogues of these organs in the invertebrata, with the view of showing how uniform is the plan on which the encephalon is constructed in the whole animal kingdom. Wherever we have a distinct *head*—that is, wherever we find the mouth placed upon a prominent part of the body, which is furnished with organs of sense adapted to the selection of the food and the direction of the movements of the trunk—there do we find a pair of cephalic ganglia, which evidently have somewhat of the same dominating influence over the whole remainder of the nervous centres, as that which is possessed by the encephalon of vertebrata. That influence, however, seems to be often much less powerfully exerted; most of the movements of the body being due to the simply reflex action of the ganglia in immediate connexion with the several organs, any one pair of which frequently surpasses in size the whole of the cephalic mass. But in proportion to the development of the organs of sense in the head, do we find the size of the cephalic ganglia increasing; and, when perfect and complex organs of vision are present, as in the higher articulata and mollusca, we find the optic nerves attaining a very large size, whilst the cephalic masses are evidently in great part to be regarded as the ganglionic centres of those nerves. This becomes particularly obvious in watching the metamorphoses of insects, as Mr. Newport long since pointed out. For there are many larvæ which have very imperfect eyes, whilst the perfect insect possesses fully developed organs of vision; and the enormous increase in the size of the cephalic ganglia, which takes place during the metamorphosis, obviously corresponds with that development. But we must not regard the cephalic ganglia of insects, &c., as entirely analogous to the optic ganglia of fishes, since it is obvious from their connexions, that they are also the ganglionic centres of other organs of sense. And as, in the higher invertebrata, the enormous development of the optic ganglia causes all the rest to be swallowed up into them (so to speak)—just as the great development of the cerebral hemispheres in man obscures many other parts of his encephalon—it is needful to go to the simpler, and, above all, to the embryonic conditions of the encephalon, to discover their real signification. Now, when the head of a myriapod is examined in an early stage of its development, as Mr. Newport has so admirably shown,* we find its encephalon in a condition so precisely analogous to that of the lower fishes, that it is impossible not to be struck with the correspondence. The head consists of two principal divisions—the *cephalic* proper, and the *basilar*,—each of them composed of four segments. Each of these segments has its pair of appendages, and each possesses its own pair of ganglionic centres. The four ganglia of the cephalic portion subsequently coalesce to form the cephalic ganglia of the perfect animal, whilst the four of the basilar portion coalesce to form the first subœsophageal ganglia. Now, the four pairs of ganglia, included in the cephalic, lie one behind the other in regular succession, like the ganglia of the encephalon of the fish, and are just as functionally distinct; for the first pair is that which furnishes the *antennal* nerves, and the second the *optic;* the third pair supplies the internal maxillæ, and the fourth pair the maxillary palpi. Thus, the cephalic ganglia of the adult animal combine the functions of three pairs of ganglia, which are connected with distinct sensory organs; and is thus obviously analogous to the chain of sensory ganglia, which makes up by far the largest part of the encephalon of the fish. There is here, as might be anticipated from the gradual disappearance of those organs in the lowest fishes, no rudiment of the cerebral hemispheres, which are at once distinguished from other ganglionic masses by their *superadded* character, having no direct connexion with any of the nerves, but being implanted, as it were, upon the summit of the strands which pass upwards from the nervous centres of the trunk. Such a rudiment seems to present itself, however, in the encephalon of the highest mollusca, the cuttle-fish tribe, which present so near an approach to fishes in the development of individual organs, although the plan of their arrangement is different; for, in addition to the highly developed optic ganglia, and the ganglia from which the arms, masticating apparatus, and other parts are supplied, there is a single cordiform mass, placed on the median line, which, from its position and connexions, may be looked upon as the nearest analogue of the cerebrum that is presented among the invertebrata. It is worthy of remark, in reference to the position in which we nearly always find the auditory ganglion of vertebrated animals (which is imbedded, as it were, in the medulla oblongata, on either side of the fourth ventricle), that the first rudiments of the *organ of hearing*, which present themselves in the gasteropod mollusca, themselves occupy this very situation; being a pair of minute sacs, containing numerous minute *otolithes* floating in fluid, which are imbedded in the posterior lobes of their cephalic ganglia, which answer to the medulla oblongata of vertebrated animals.

Quitting, now, these forms of the nervous system, which represent the encephalon of the vertebrated animal deprived of its cerebrum, let us briefly examine into the nature of those more complex forms, which are characteristic of the higher vertebrata. It is not enough to pass at once from the encephalon of the fish to that of man. The true character of *his* brain cannot be known without a patient and gradual survey of the whole ascending series, nor without a careful comparison of the gradations

* Linnæan Transactions, vol. xix; see also our Twentieth Volume p. 493 et seq.

thus encountered, with those which are presented to us in the progress of embryonic development in the most perfect and complex form. A very cursory examination yields to us this important deduction,—that the increased complexity of the encephalic structure, in the higher vertebrata, is almost entirely due to the extraordinary development of the cerebral hemispheres, and of their commissural connexions with the other ganglionic masses; the development of the sensory ganglia not making by any means the same proportional advance, or rather, advancing rapidly up to a certain point, and then ceasing. A new and special series of observations are much required, for the sake of determining the relative sizes of the several organs we have indicated in different animals, both with respect to each other and to the bulk of the body. Thus, it is well known that the general statement, that the brain of man is larger in proportion to the entire weight of his body, has been disproved by the fact that a higher proportion is presented by several small birds and rodens. But if the statement had been made, that the *cerebrum* of man is heavier in proportion to the weight of his body than that any other animal, we apprehend that it would have been strictly correct. For the encephalon of birds is still made up chiefly of the chain of ganglia we have described, which attains, we believe, its highest development, relatively to the weight of the body, in that class. On turning aside the cerebral hemispheres (which do not yet altogether cover in the optic lobes), we find that they really form but a thin lamina, beneath which lies a series of large protuberances, which the study of their connexions at once discovers to consist of the corpora striata, thalami optici, and optic ganglia. If these were weighed, exclusively of the cerebrum proper and cerebellum, we are assured that they would bear a larger proportion to the whole body of the smaller birds, than they would in any other class.

Before passing from this subject, we must notice an extraordinary error, into which Mr. Noble has been led whilst criticising certain opinions of Dr. Carpenter's, in reference to the functions of the corpora quadrigemina of man. "In birds," he remarks, "the optic tubercles (the *nates*) alone are found; the certain analogues of the *testes* do not exist;" whence he infers that Dr. Carpenter must be wrong in attributing to these organs any share in the instincts and emotions, having demonstrated to his own satisfaction that such a class of functions must be limited to the *testes*, if appertaining to the corpora quadrigemina at all. And, as birds have instincts and emotions, but no *testes*, Dr. Carpenter must be wrong in his allocation. Now we shall hereafter discuss the general question of the connexion of the instincts and emotions with the sensory ganglia, and shall only stop to point out the fallacy of Mr. Noble's argument. The *corpora bigemina* of birds are actually *far larger* in proportion than the *corpora quadrigemina* of mammals; and although the duplex enlargements of the latter might lead to the idea that these organs receive an increased development in that class, yet there is no proof whatever that the peculiarity is anything but superficial, the connexions of the optic ganglia in mammals and birds being exactly alike. We shall presently find that the *very large* proportional size of the optic ganglia in birds is an important argument in favour of the very doctrines of which Mr. Noble, (doubtless owing to having derived an erroneous notion of them, from the imperfect manner in which they were first expressed by Dr. Carpenter) has given, in sundry parts of his work, a very strange misrepresentation.

The development of the cerebral hemispheres in the ascending vertebrated series, and in the human embryo, has lately been elaborately investigated by Professor Retzius of Stockholm; and he has given the most complete confirmation of the statements of former comparative anatomists in regard to the order in which the different lobes of the cerebrum are evolved, whilst he has shown that the same order prevails in the embryological development of the human encephalon. Notwithstanding the contradiction of Mr. Noble and the phrenological luminaries whom he quotes, we rely upon the testimony of those who *have* studied Nature and put her to the question in this matter, for the universal truth of that which a very limited amount of observation serves to indicate; namely, that the part of the cerebrum which is *most* developed in man, in comparison with other animals, is not the anterior but the *posterior*. This was long ago asserted by the opponents of Gall, upon the ground that the cerebrum of none of the lower mammalia extends so far backwards as to cover the cerebellum; and the objection was met by the phrenologists with the assertion, that there is no anatomical division between the middle and posterior lobes, and that it is quite conformable to our general knowledge of the disposition of the several parts of the encephalon to suppose, that the parts corresponding to the posterior lobes of man do exist in the cerebrum of the lower mammalia, but that they are not extended so far backwards, because the whole mass of the cerebrum in man is pushed backwards by the increased development of the anterior and middle lobes. Now although there is no external line of separation between the middle and posterior lobes, such as exists between the anterior and middle, there are two points of internal structure which afford as definite an indication as can be required; these are the development of the posterior cornua of the lateral ventricles, and the situation of the hippocampus major. By these means it may be unequivocally determined, that the development of the *posterior* lobe of the human cerebrum is far greater in proportion to the whole mass, than it is in the highest of the other mammalia; and that in the lower mammalia, as in all inferior vertebrata, *the posterior lobe is totally wanting*. Further, the philosophical anatomist well knows that the rudiment of a cerebrum which exists in fishes, represents the *anterior lobe only;* that this enlarges as we ascend through the classes of reptiles and birds, but does not change its character; that the middle lobe is only developed as we enter the mammalian class, presenting itself at first in a very rudimentary form, and attaining increased development as we ascend; and that the posterior lobe is developed from the back of the middle lobe, making its first appearance in the carnivorous group.

This history *finds an exact parallel in that of the embryonic development of the human cerebrum;* the rudiment which presents itself at a period when the chain of sensory ganglia has attained an advanced development, having been shown by Professor Retzius to be the representative of the anterior lobe only, the development of this making considerable progress before the middle lobe begins to be evolved, and the posterior lobe being the latest in order of evolution. Strange as this assertion may be to phrenologists, we have, nevertheless, an implicit confidence in its truth, arising from the knowledge which personal acquaintance has lately enabled us to form, of the remarkable perseverance and sagacity with which the eminent professor of Stockholm pursues his investigations, and of the habits of philosophical deduction which he exercises in drawing conclusions from his observations. The following summary of these we extract from the Monthly Report of the Royal Academy of Sciences at Stockholm, to which they have been recently communicated. We may mention that they fully coincide with the observations made by Tiedemann thirty years since ; which were, however, neither so precise nor so complete.

"In the first period, which corresponds with the second and third months, only the anterior lobes form ; in the second period, which is comprised in the end of the third month, in the fourth, and in a small portion of the fifth, the two middle lobes appear ; and after this time the posterior lobes. During the first period the descending horns of the lateral ventricles and the pedes hippocampi are wanting: these are added in the second period. During a great portion of the first period the hemispheres do not cover the thalami nervorum opticorum ; in the second period they completely overlap these parts, approach the large corpora quadrigemina, cover their anterior part, and then descend by the side of the cerebral nucleus (cone or stem), and, as it were, fold round it. If we examine a brain at this period of development we might, from its external appearance, imagine that the posterior margin of the hemispheres corresponds to their persistent posterior ends and margins, i.e. to those which are their posterior margins in their perfectly developed state. But it is not so. If we open the brain we come at once to the descending horns of the lateral ventricles, in which are the rudiments of the great pedes hippocampi. At a later period, in the fourth month, a small superficial notch is formed at the posterior margins of the hemispheres ; and that part of this margin which is above the notch is the first rudiment of the posterior lobes of the hemispheres. These, which are thus for a long time only rudimental, begin above the middle lobes, gradually take in their posterior margin, follow it down, as development advances, by the sides of the cerebral nucleus, and terminate at that part of the middle lobes which meets the pedes hippocampi. Even in the brain of the mature fœtus, as well as in the fully developed brains of older persons, the posterior lobes are very clearly separated from the middle lobes by a branching furrow, which is especially distinct, on the vertical side of the hemisphere which lies next to the falx."

It is interesting to observe that these researches find a parallelism in another sub-kingdom ; for they coincide as to the direction of the development with the account which has been already given by Mr. Newport, in the Linnæan Transactions, of the manner in which the *cephalic* portion of the head is developed in the myriapod and other articulata, by an extension of the coalesced segments of which it is formed, from before backwards, so as to overlap the basilar portion, in exactly the same way as the cerebrum is now shown by Professor Retzius to overlap the corpora quadrigemina and cerebellum.

Now we affirm that no phrenologist is entitled to dispute such a position, without having followed up a similar investigation with similar perseverance and with equal capacity. And at present the explanation to which we just now alluded, as having been offered by the followers of Gall, is capable of being turned pointedly against themselves ; for if, as they maintain, the increased development of one lobe of the brain may cause the displacement of another, the fullness of the *forehead* in man may be attributed to the development, not of the *anterior* lobes of his cerebrum, but of the *posterior,* these being the last to be evolved, and the parts whose superior development is most marked in him.

Following out our principle of endeavouring to determine, in the first instance, the functions peculiar to divisions of the nervous centres, whose "mechanical distinctness" is obvious, we shall proceed to inquire into the relative endowments of the cerebral hemispheres, and of the ganglia of sensation: and shall investigate the evidence afforded on this subject; first, by comparative anatomy; and, secondly, by experimental inquiry. Now the first remark that is forced upon us, in the most cursory examination of the structure of the cephalic ganglia of different animals, is this; that the *essential* part of the encephalon is formed by the collection of sensory ganglia, and that the cerebrum is an organ *superadded* for some purpose, which is only fully attained in the higher vertebrata. We see the cerebrum dwindling away as we descend the scale; until, in the lowest of the class of fishes (the amphioxus, or lancelot) no rudiment is left of it. But we find no such corresponding diminution in the sensory ganglia, even in those which have been commonly most regarded in the light of appendages to the cerebrum. And on proceeding to the invertebrata, we find the absence of the cerebrum to be the almost invariable rule, even where the sensory ganglia attain their highest development. We never find a cerebrum without the sensory ganglia; but, *in by far the greatest number of animals, we find the sensory ganglia without the cerebrum;* and it is only in mammalia, that the development of the cerebrum is such, as to give to it any obvious predominance over them.

We think ourselves fully entitled, then, to infer from these facts, that *the cerebrum is not the seat of sensation;* since we must otherwise believe that those animals which are destitute of it—insects, for example—are destitute of sensibility. In like manner, we may infer that the several classes of impressions are communicated to the consciousness through the medium of the ganglia, in which the nerves that convey them terminate; the optic ganglia, for instance, being the seat of visual sensations, the auditory, of the sensations produced by sounds, and so on. For there is no single organ that seems to be sufficiently the centre of the rest, for us to regard it as the special sensorium. Guided by this view, it seems to us not difficult to assign a function to the thalami optici; for whilst we have distinct olfactive, optic, and auditory ganglia, it seems but reasonable to expect, that we should have a ganglion for the formation of the sensations communicated by the nerves of touch; and the connexion of the thalami with the spinal cord into which all these nerves pass, is precisely what we should anticipate on such a view of its offices. Moreover it is interesting to remark that the connexion between the thalami and the proper optic ganglia is everywhere very close; and this, again, harmonizes well with the fact which is familiar to every one, that the sensations of sight and touch are more closely associated, both in the origination of mental *perceptions* (or general notions of the characters of the objects of sensation) and in the direction of muscular movements, than are any other pair of sensations.

But we think it may be further asserted with confidence that the ganglia in question are the sources of all those movements, which, following directly upon sensations, may be termed *consensual*. This is a class of actions which has, we think, been too little scrutinized by physiologists; and we are much mistaken if, under the guidance of comparative anatomy and experiment, combined with pathological observation, the subject is not capable of considerable elucidation. We must content ourselves with here briefly indicating the outline of the doctrines, which have been long in progress of development in our own minds, and which have now attained a completeness and consistency, that warrants our expressing them with some degree of confidence. Putting aside the purely reflex actions, in which a movement is directly excited by an *impression*, without the intervention of a *sensation*, a little observation of our own corporeal phenomena will bring under our notice many instances, in which movements are excited with equal directness and constancy by *sensations*, without the intervention of anything that can be properly called an *act of mind*, the simple *consciousness* being alone requisite. We may take, as illustrations, the closure of the eyelids to a dazzling light, the sneezing which is excited by the same cause, the start produced by sudden loud sounds, the vomiting induced by certain nauseous tastes or odours, and the movements of the respiratory muscles (as in laughter) which are brought on by the sense of tickling. We have purposely adduced examples of the class of movements in question excited through each of the senses, in order to show how universal is the principle for which we are contending. Knowing, as we do, that the sensory ganglia not only receive the sensory nerves, but are connected, by their implantation on the fibrous tracts of the medulla oblongata, with the motor system, we can at once understand the channel through which sensations should thus produce movements, without involving any higher act of the mind, or any exertion of the will. In the case of common or tactile sensation, there seems good reason for regarding the corpora striata as the motor portion of the ganglionic mass, of which the thalami optici constitute the sensory; the relation between them being, as well pointed out by Messrs. Todd and Bowman, the same as that which subsists between the anterior and posterior peaks of vesicular matter in the spinal cord.

Now if we inquire what movements in the lower tribes of animals are analogous to these consensual actions in ourselves, we think that a little consideration will make it at once apparent, that we are to refer to this category all those movements which are purely *instinctive*. We have on a former occasion pointed out the general characters, by which these actions may be distinguished from the volitional (Vol. XI, p. 96); and we need here only refer to their constant and immediate dependence upon sensations, and the absence (so far as we can at all discern) of anything like *an idea* of the purpose, towards which their operations are directed. We can, in fact, discover no middle view that can be taken, between the supposition that the purely instinctive actions (in which category we must include nearly the whole of those performed by insects) are immediately and necessarily prompted by sensations; and the notion that the sensations excite ideas, that the ideas are made the subject of reasoning processes, and that these reasoning processes issue in an effort of the will,

*designedly directed* (as in almost every action of adult man) towards the attainment of the object. Now if this be the case, it leads us to an important view respecting the mechanism, so to speak, of instinctive actions. Our usual method of reasoning upon these subjects leads us to class these actions as manifestations of different instinctive propensities; and a learned entomologist has enumerated between thirty and forty instincts which must be attributed to the bee. Upon phrenological principles all these ought to have their separate location in its minute cephalic ganglia. But we fearlessly assert that this classification of the several actions in question, as manifestations of different propensities at all corresponding with those of which we are conscious in ourselves, is entirely of our own making, and has no real existence in any brain but our own. We might as well expect to find a distinct organ in our own encephalon, for the consensual movement of the eyes, for laughter, sneezing, or any other association of muscular actions called into play by the direct agency of sensation. The sensory ganglia are, evidently, taken as a whole, the instruments by which the instinctive or consensual actions are performed. But why should we suppose different parts of the optic ganglion to be concerned, when the sight of an object of food prompts the movements requisite to obtain it, and when the sight or sound of one of the opposite sex calls into action the muscles, that bring the insect into close relation with it? What is needed is a ganglionic mass for the reception of sensations and for the origination of muscular movements; *what* movements shall follow *what* sensations, must be determined, according to the expression of Prochaska, by the "laws written upon the nervous pulp," just as in the case of reflex actions. We see no reason to doubt that insects and other animals, whose actions are chiefly instinctive, possess feelings of pain and pleasure in connexion with particular sensations, resembling those which *we* experience in connexion with the sensations that excite consensual actions in ourselves. But we can find less ground for the belief that they possess feelings of *desire* for any objects, analogous to those which form so important a part of our own mental operations. For these feelings result from a distinct *idea* or *conception* of the object; and their purpose is, evidently, to prompt to the reasoning processes which are required for its attainment. We do not *feel a desire* to close our eyes against a strong light, unless our attention be directed to the matter, and we attempt to resist the natural action which is prompted by the sensation. We do not *feel a desire* to start on a loud aud sudden sound; on the contrary, the action *necessarily* and immediately results from the sensation, unless we have expected the sound, and put the will strongly in action to resist the impulse. We do not *feel a desire* to vomit when we taste or smell anything nauseous, for our desire is naturally the other way, and nothing but the strength of the sensation can overcome it.

This class of phenomena receives a remarkable illustration in certain morbid conditions of the system. Every one of our readers must have witnessed cases of unusual susceptibility to light, sound, tactile impressions, &c., evidently originating, not in disordered conditions of the organs themselves, but in affections of those parts of the sensorium, which are the centres of their respective nerves. A very instructive case of this kind, in which a variety of curious movements were directly excited by sensations, has lately been recorded by Dr. Cowan.* We trace the same influence in hydrophobia, in which the convulsive paroxysms are brought on by the *sight* or the *sound* of liquids. And we have another very instructive illustration, in the act of yawning, which, when the encephalic centres are in a state of preparation, induced by previous fatigue, is excited simply by the *sight* or the *sound* of the action in another person, without the attention being at all directed to the sensation, or any idea being attached to it. Upon phrenological principles, this action must be referred to a propensity to imitation, which has its local habitation in the cerebrum; but we cannot ourselves perceive any reason whatever for regarding it in any other light than as a consensual action, in which the optic or auditory ganglion (as the case may be) is alone concerned.

Our location of the centre of the actions of this class, in the cephalic ganglia, to the exclusion of the cerebrum, has been made upon grounds derived from comparative anatomy alone; but we shall find it completely borne out by the results of experimental inquiry upon the higher vertebrata. In addition to the general observations which we have already made upon this subject, we think it well to add one or two remarks upon the special applicability of experiment, to the determination of the functions of the encephalic centres. In the first place, we freely admit that no good purpose whatever can be derived from any hap-hazard incisions or slicings, or from the partial removal of any single organ. But we do affirm that, if we take the guide which Nature affords us, and remove *entire* organs, whose distinctness she herself indicates to us in language not to be mistaken, we may obtain important information, confirmatory of that which we derive from other sources. But it is to be steadily kept in view, that here, as in other instances, when we bring together and compare the results obtained by different inquiries, we must be careful to separate the *phenomena* which actually presented themselves, from the inferences drawn from them by those who have recorded them. By so doing, we shall be able to reconcile many apparent contradictions; and shall find a much greater amount of accordance, than was to be anticipated from a cursory survey of the apparently discrepant accounts. Of this we shall presently have a striking instance, in reviewing the results of experiments upon the cerebellum. Moreover, we must draw a marked line of distinction between the *positive* and *negative* value of the evidence we accept. If we find that, after the removal of an entire organ, some function supposed to depend upon it still exists, with little or no impairment, we have a right to say that the function *cannot* be dependent upon that organ. On the other hand, the evidence afforded by the disappearance of a certain function or power, after the removal of any particular division of the nervous centres, is less satisfactory, and needs to be sifted with great care. But we are not entitled to reject it as unworthy of consideration, because we find the results in some degree discordant; still less if we find that, after the repetition of such experiments by a considerable number of independent vivisectors, the phenomena themselves present such an accordance, as cannot be accounted for by the general disturbance of the functions of the nervous centres, produced by the severity of the lesion itself.

* Lancet, 1845, vol. ii, p. 364.

When the cerebrum of one of the higher vertebrata is entirely removed, the animal is reduced to the level of one of the invertebrata, which is naturally destitute of the organ; and it is important to ascertain, if the actions which it then performs are of the same nature as those, which are so characteristic of the latter. But, in judging of the results of such vivisections, it is important to bear in mind, that the functions of the remaining organs cannot but be impaired by the removal of a large part of the encephalic mass; and also that these functions, in the unmutilated animal, are by no means so obvious in its actions, where a cerebrum is present, as where it is altogether wanting. Thus, whilst nearly all the actions of insects, which are not reflex, may be regarded as the direct results of the exciting sensations, the actions of birds, whilst presenting a general similarity with them, are more obviously influenced by intelligence, and are, therefore, for the most part referrible, not so much to the consensual group as to the volitional. The variability of the actions of different individuals of the same species, under similar circumstances,—the actions themselves all tending to the same end, which is suggested and kept constantly before the mind by the prevailing sensation,—becomes very striking in the class of birds, when we closely scrutinize their habits, and contrast them with those of the other class, to which they bear so remarkable a general analogy. Making such allowances, then, we think that experimental inquiry affords satisfactory evidence of the identity in the condition of a bird or mammal, whose cerebrum has been removed, with that of an insect or other invertebrate animal which naturally possesses none. In the first place, we think it impossible to analyse and compare the results obtained by different vivisectors, without coming to the conclusion that *sensibility*, both of a general and a special kind, remained after the removal of the cerebrum. Doubtless there are fallacies attending the deduction of any such inferences from the movements performed by the animal under various stimuli; since a large proportion of these movements may fairly be set down as of a reflex character, and as not indicating sensation. It is safer, therefore, to draw our conclusions from movements consequent upon such impressions made upon the organs of *special* sense, as cannot (so far as we are aware) excite movements without the intervention of consciousness. And it appears to us to be an inevitable deduction from these phenomena, that the animals retained the possession of the senses of sight and hearing, though in a very imperfect manner. It is not a little curious that M. Flourens, who at first drew a contrary inference from his experiments,—maintaining that the removal of the cerebrum destroys all sensibility,—should have since abandoned this opinion, in favour of the one put forward by Cuvier and his colleagues, in their well-known report upon M. Flourens' first Memoir; having substituted, in the second edition of his 'Experimental Researches,' the term *perception* for *sensation*, whenever he speaks of the function which is destroyed by the removal of the cerebrum.—Secondly, in animals deprived of the cerebrum, consensual movements were excited by the sensations. Thus, a bird would follow the light, and it would avoid objects placed in its way; it would start from its nocturnal sleep, raising its head and opening its eyes, upon the slightest noise; and it spent a great part of its time in pruning its feathers and scratching itself. Even if we regard the last of these actions as reflex, the preceding can scarcely be so considered. Making a fair allowance, then, for the disturbance in the condition of the whole encephalon, which is necessarily induced by the removal of so important a part, and for the small proportion which the simply consensual actions bear in the general movements of these animals, we think ourselves entitled to affirm, that the conditions essential for these actions remain after the removal of the cerebrum, and that we are not to regard that organ as having any necessary participation in them.

Other experiments prove how much the ordinary movements of birds and the lower mammalia are *directly influenced* by sensation. Thus, vertiginous movements may be induced in pigeons and rabbits, not merely by destroying one of the optic ganglia, but by simply blinding one eye, either by evacuating its humors, or by temporarily bandaging it. And we believe that the similar movements produced, in the curious experiments of M. Flourens, by lesions of the semicircular canals, are readily explicable upon the same principle,—perverted sensations in regard to the direction of sounds (of which it seems to be the special object of that part of the auditory apparatus to take cognizance) being thus communicated to the sensorium, and producing abnormal movements, the direction of which bears a constant relation to that of the canal. These and other similar experimental results, which are referred to by Mr. Noble with a sort of contempt, as not affording us the slightest knowledge of a precise kind in regard to the functions of the different parts of the encephalon, appear to us, on the contrary, when surveyed from the point of view to which we are led by comparative anatomy, to be in admirable harmony with the conclusions indicated by that mode of investigation.

Although the development of the cerebrum in the vertebrata appears to *supersede* the simple and direct actions of the sensory ganglia and the motor fibres proceeding from them,—and this, in a degree comparable to its superiority of size,—it by no means *alters* their function. The simply consensual movements gradually form a smaller and smaller proportion of the whole actions of the animal as we ascend the scale; just as, in the invertebrata, we find the simply reflex operations of the lower gradually brought under the guidance and control of the consensual, which are so characteristic of the higher. The very same gradation may be traced in the human infant. No one can watch its actions with an educated physiological eye, without perceiving, that they are at first almost purely reflex; the nipple of its nurse being seized by the lips when they are applied to it, and the act of suction being performed, as Mr. Grainger's experiments prove, under circumstances that forbid any other idea being attached to them. But we soon find that it is guided to the nipple by the sense of sight, and also (we think) by that of smell, which appears to us to have much to do with this action; and the first movements which it performs, when it begins to take notice of surrounding objects, are evidently to be referred to the simply consensual group. This is just what we should expect from the fact, that the spinal cord and the sensory ganglia at its summit are the parts of the nervous centres which are first developed, and which most rapidly advance towards maturity. Their actions are sufficient for the human infant for a long period after its birth; and it is not until it is several months old, that we can discern any, which can be unequivocally attributed to a dawning intelligence. From the time when this manifests itself, the simple consensual movements become less and less obvious; and at last they escape our notice altogether, if we do not carefully scrutinize our own actions. But it does not hence follow, that the sensory ganglia and their motor nerves are thrown out of use; or that their only function is to receive sensations and impart them to the cerebrum, without having any concern in the movements which it originates. For we think that all recent observations and experimental inquiries tend towards this conclusion—that *the cerebrum has itself no direct connexion either with the sensory or motor nerves;* and that as it receives sensations through the medium of the various ganglia, in which the sensory nerves terminate, so it executes the mandates of the will through the motor fibres which originate from those same ganglia, or from others in immediate connexion with them. The belief has long prevailed, that the motor power of the cerebral hemispheres is chiefly exerted through the corpora striata; and we have already proved, from the comparative anatomy of these organs, that they are by no means to be regarded as mere appendages to the hemispheres, but are independent ganglia, of which the cerebrum makes use (so to speak) to perform its movements. Bearing in mind their intimate connexion with the thalami optici, we think it probable (as already remarked), that they constitute the motor portion of the ganglionic centre of the tactile sensations and respondent movements; and, if this be the case, the reason of its peculiar connexion with the cerebrum at once becomes apparent. For all voluntary movements require *the guidance of sensations;* and most of these are of the tactile kind. The "muscular sense" of Sir C. Bell is a necessary condition of our keeping up any voluntary contraction of a muscle, unless some other sense is substituted for it,—as in the case of the woman, affected with anæsthesia, who could hold her child upon her arm as long as she looked at it, but could not, by the strongest effort of her will, sustain it after withdrawing her eyes. In the movements of the eyeballs, the guiding sensations (as Dr. Alison has shown) are ordinarily those received through the sight; and in the movements of the larynx and muscles of articulation, the guiding sensations are ordinarily those received through the hearing; but, in either case, if these senses are wanting, a sufficient amount of guidance seems to be derivable from the muscular sense. No instances occur to us, in which voluntary muscular movements are dependent upon the guiding sensations, derived from the smell or the taste.

Thus, then, the spinal cord with the ganglia at its summit,—forming altogether what may be termed the *cranio-spinal axis,*—to the exclusion of the cerebrum and cerebellum, furnishes all the conditions for the reception of sensations, and the performance of muscular movements; and the two latter organs may be regarded as superadded for some special purpose, receiving their stimulus to action through the medium of the cranio-spinal axis and its nerves, and exerting their influence on the muscular system through the same channel. In adopting this view, which is by no means a novel one, we think that we can harmonize completely all the results afforded by anatomical research and by experimental inquiry. It corresponds particularly well with the varying intimacy of the connexion between the cerebrum and the different ganglia at its base, and with the relative development of the latter. For the connexion is *most* intimate with the thalami optici and corpora striata, which we regard as together constituting the ganglionic centre of tactile and muscular sensations, and of the respondent motions ; and these ganglia are in man by far the largest of the whole series ; both of which facts seem to correspond with the great predominance of the movements guided by these sensations over all others. Next, in order of intimacy of connexion with the cerebrum, and also of size, come the optic ganglia or tubercula quadrigemina ; and this again corresponds with the fact that, next to the tactile sensations, the visual are of most importance in regulating the muscular movements. The auditory ganglia, lodged in the substance of the medulla oblongata, are of smaller size, and have a less intimate connexion with the cerebrum ; in accordance, as it would seem, with their inferior concern in the regulation of the voluntary movements. But they are in immediate relation with the central connexions of the nerves exciting the actions of the larynx and of the muscles of articulation ; and thus serve the important purpose, as we have already pointed out, of regulating the movements of the organs of speech. And the olfactive ganglia, of which the ganglionic matter in man is present in comparatively small amount, are the most removed, of all the ganglia of special sense, from intimate connexion with the cerebrum ; and they are the least concerned in the direction and regulation of the voluntary movements. In regard to the sense of taste, we need only remark that (as most of our readers are probably already aware) there is strong reason for regarding it as only a modification of that of touch ; and if it has a proper ganglionic centre, this will be a portion of the vesicular nucleus of the medulla oblongata, in which the sensory division of the fifth pair, and the glosso-pharyngeal nerve find their termination. This position, too, would correspond with the slight participation which the sense of taste possesses in the regulation of the voluntary muscular movements ; those of the tongue, in the examination of a sapid body, being the only ones which we can attribute to it.

Before we quit the subject of the sensory ganglia, we must remark that they appear to us to be the seat of the simple or elementary *feelings* of pleasure or pain, which are connected with different sensations. If we deny this, we must deny the existence of such feelings in animals whose encephalon consists entirely of sensory ganglia ; which, we imagine, few will be disposed to do in regard to insects, to say nothing of inferior tribes. We shall presently attempt to show that, in animals possessing a cerebrum, these feelings may be associated with *ideas*, the impressions made by which are transmitted from it *downwards* to these ganglia ; as well as with the *sensations*, whose impressions are received in an upward direction from the sensory nerves.

We now come to consider the general functions of the cerebrum ; in which determination we are guided by subtracting from the sum-total of the functions of the nervous system all that it can be ascertained *not* to do. It would be interesting, if we had space to dwell upon such a topic, to trace the gradual limitation which has taken place in the physiological idea of the functions of this organ; from the period when it was considered as the centre of the whole life of the body,—the elaborator of the "animal spirits," whose circulation through the system by the nerves was regarded as essential to every vital action; to the time of our present writing, in which the real extent of its agency is so much better understood. The withdrawal of the organic functions from its support or control, was the first step; the proof of the independent endowments of the spinal cord was the next; and the persistence of sensibility and of the power of consensual movement, after its removal, was the third great advance in this limitation. The cerebrum, then, has no concern in the purely excito-motor actions. It is *not* the sensorium, or instrument, by which the mind becomes conscious of sensory impressions. Nor does it participate in the performance of the movements, which are directly connected with these sensations. Nor does it seem necessary for the feelings of pain and pleasure, which appear inseparably connected with them. What then is its office? In the first place we may attribute to it the formation of those *notions* or *ideas* respecting the objects of sensation, which are the materials upon which all the higher processes of mind operate; the power concerned in which process, the process itself, and the result of it, are all known by the term perception. We may attribute to it, also, that power of recording sensations and the ideas connected with them, which we call memory; and that power of recalling them, which is designated conception. Now it is easy to understand, that the direct communication between the cerebrum and the sensorium should cause the ideas excited in the former by sensations, to produce feelings of pleasure or pain in the latter, just as do sensations themselves; and the same result will take place from the recall of past sensations and ideas, or the conception of those which are future, founded as this is upon the experience of the past. We lay much stress upon this view, that the *ideas* are *cerebral,* whilst the *feelings* of pleasure or pain connected with them are *sensorial,* for reasons that will presently become apparent. Its correctness seems to us borne out by a variety of considerations. We cannot see any reason for supposing, that there is a different seat of pleasure or pain for a remembered or anticipated sensation, from that in which these feelings are aroused by an actual sensation. The intermediate position of the sensory ganglia, receiving as they do the fibres from the sensory organs on the one hand, and from the cerebrum on the other, points them out to us as the seat of *consciousness,* not only of what is taking place in the *external world,* but also of the *operations of the cerebrum itself*; and if of consciousness, then undoubtedly of the pain or pleasure so immediately connected with it. But we have a stronger ground than this, namely, that the very same consensual movements may be excited by remembered or conceived sensations as by the actual ones; showing the exact analogy between the downward action of the cerebrum, and the upward action of the sensory nerves, in exciting changes in the sensorium. We might cite numerous examples of this kind; the following will suffice. In persons with irritable stomachs, the act of vomiting may be excited by an offensive smell, a nauseous taste, or a disgusting sight; and the remembrance or conception of similar sensations will produce the same effect. When the nervous system is in the condition, that renders the act of yawning easily excitable, it may be induced, not merely by the sight or sound of yawning in another person, but by the mere *idea* of it, called up by the mention of the term. In like manner, in hydrophobia, the *idea* of liquids, called up intentionally or accidentally, is frequently as efficient in exciting convulsive paroxysms, as the actual sight or sound of them.

These considerations appear to us to lead to a view of the nature of the propensities and emotions, which is different from that ordinarily entertained, and which seems to us to harmonize better with our knowledge of mental operations. As we have already noticed, we can only judge of these propensities in the lower animals by the actions they perform; and we are likely to be in great error, if we always attribute to them the same desires or emotions as we ourselves experience, when we execute similar actions. For these desires or emotions involve *an idea* of the objects of them, such as is formed in ourselves for the purpose of stimulating to action the reasoning powers and the will; and in animals which have no reasoning powers, and in which the action is immediately prompted by the sensation, there is no reason to believe that such ideas or notions are formed. Let us take, for example, the sexual propensity. This, in the human being, may take the form of a desire, excited by sensations, some of them internal and some of them derived from without; and this desire stimulates the reasoning powers and the will to the operations necessary for the indulgence, or, it may be, for the restraint of that desire. But in the acts immediately preceding the *emissio seminis* (which is itself reflex) we trace the *direct* operation of sensations; and this appears to be the whole history of the process in those tribes, whose instincts are not restrained or directed by intelligence,—*all* the movements concerned being the direct result of the exciting sensations, in other words consensual. We may easily, of course, believe that the sensations are painful, when sexual intercourse is prevented, as they are pleasurable when it takes place; but we affirm that there can be nothing like a desire, unless there be a capability of forming an idea of the object of that desire, which the sensory ganglia alone are unable to do. Instead, then, of being that simple propensity which some would regard it, the so-called amativeness is of a complex nature. In so far as the action referred to it is simply consensual, the abstract principle (as we have already shown in regard to the propensities in general, p. 504) has no real existence. And in so far as the propensity has the form of a desire, of which the mind is conscious, this desire is resolvable into the formation of an idea or train of ideas, which the mind feels it pleasurable to entertain. The first operation, the formation of the idea, is purely *intellectual;* the second, the feeling of pleasure connected with it, which is the cause of our continually dwelling upon it, and of its powerful operation upon the reasoning processes and will, appears to us evidently *sensorial.* In certain disordered states of the nervous centres, the sexual sensations may be so highly excited, that they act directly upon the muscular system, not only without the aid of the intellect and will, but even in opposition to it, as is seen in the violent semi-convulsive movements, excited in a nympho-maniacal patient by the sight of one of the opposite sex.

In like manner, we might analyse the propensity to imitation; and might show that it is partly sensorial, partly intellectual. Thus there is some imitation, which is altogether consensual; the sensation, in each case, immediately exciting the movement. This is the mode in which infants learn to perform many of the actions which they witness in adults; and in which many adults are led to imitate involuntarily, and even unconsciously to themselves, under circumstances where they would least wish to do so. Take, for example, the case of yawning, before referred to; in which the operation of this consensual power is peculiarly evident, as it is impossible to execute a thoroughly good yawn by an act of volition alone. Now it seems absurd to refer such an action to an abstract principle of imitation, and to attempt to localize this principle; when we can trace a connexion as direct and immediate between the sensation and the muscular movement, as we can between the impression and the respondent action in the case of a movement excited through the spinal cord. On the other hand, there is a propensity to voluntary imitation,* as shown in a fondness for mimicry, &c.; and this, like the amative desire, is resolvable into the intellectual *idea* and the *sensorial pleasure* accompanying it.

In like manner we might analyse all the propensities, emotions, and moral feelings; between which and the intellectual operations, phrenologists would establish such a marked distinction. What is benevolence, for example, but pleasure in the contemplation of the happiness of others? What is the whole class of selfish propensities, on the other hand, but the feeling of pleasure in the entertaining of various ideas connected with self? What is combativeness, but the pleasure of setting one's self in antagonism with others? Or what is veneration, but the pleasure of contemplating rank or perfections superior to our own? What is hope, again, but the pleasure derived from the anticipation of future enjoyment? Or what is cautiousness, but a mixed propensity, resulting from fear or anticipated pain, (to which phrenology does not assign a separate organ,) combined with pleasure in the contemplation of precautions adopted to ward it off? The connexion between the elementary feelings of pleasure and pain, and the operation of the various propensities, is universally admitted; the usual explanation of this fact is, that the indulgence of the propensities is a source of pleasure, whilst the restraint of them is attended with pain. Now, on the other hand, we consider a proper analysis of the processes in question to show, that the propensity cannot exist without a distinct *idea* of the objects to which it is directed; that the existence of such ideas, like that of the sensations from which they originate, is attended with pleasure or pain; and that, according to the respective degrees, in which these feelings attend particular classes of ideas, (as to which there is obviously a difference among individuals, analogous to that which exists with regard to the feelings of pleasure or pain excited immediately by sensations,—the likes and dislikes of different articles of food, for example,) will be the disposition of the mind to entertain them, the frequency with which they are brought before it, and the influence they are allowed to exercise in the formation of our voluntary determinations.

We believe, then, that Dr. Carpenter is perfectly correct in maintaining

* In this manner we can understand a fact, of which, though sufficiently common, we never met with a professed phrenologist who could give us an explanation,—that the tendency to involuntary imitation may exist very strongly in persons who are utterly unable to mimic or imitate voluntarily, and *vice versâ.*

that the offices of the cerebrum are restricted to *intellectual* operations ; understanding, by that term, the operations which are concerned in the formation of a voluntary determination ; and the movements consequent upon which are expressly adapted to an end distinctly conceived in the mind of the individual. But this allows full weight to the operations, through the instrumentality of the cerebrum, of what are commonly termed the emotions, as the *active principles* in the guidance of our conduct ; since, as just shown, the emotions are not simple but composite states, the formation of ideas being the part of them that is performed by the cerebrum, whilst the sense of pleasure or pain, which is the real *feeling* connected with them, has its seat in the sensory ganglia. And the objection, brought by Mr. Noble and Mr. G. Combe against Dr Carpenter's views,—that the most active principles of the mind, the mainsprings of the greater number of intellectual operations, are thereby located in a portion of the nervous centres, whose size bears no relation to their importance,—falls to the ground when those views are thus qualified. In the form, in which we have now endeavoured to develope them, they fully explain the mode in which emotions, directly excited by sensations, or recalled by an act of memory, may act upon the nervous system, in such a manner as to produce movements, which themselves belong to the consensual group. We may take the case of laughter as an illustration. The consensual nature of this action is proved by the facility with which it may be excited, in most persons, by the act of tickling ; this peculiar impression (conveyed through the ordinary spinal nerves) being competent to excite reflex actions in the part itself, and in other muscles, through the medium of the spinal cord; but only having the power of exciting laughter, when it reaches the sensorium and is *felt*. In like manner the action may be directly excited by sights or sounds, to which no idea is at first attached, and which do not therefore operate by calling up an emotional state. We say that such things are ludicrous, when we come to consider them, simply because they excite involuntary laughter; and we can give no further account of them: and, as in the case of pleasure or pain connected with particular sensations, a sight which provokes one person to laughter may be perfectly inert as regards another. In many cases of this kind, the *will* is strongly exerted to check the consensual movement; successfully or otherwise, as the case may be. The character of the action is further shown by observation of infants, in whom smiles and laughter obviously result from the stimulus of sensations, before they can have any ideas connected with them. But the more ordinary cause of laughter in the adult is a ludicrous *idea*, which is aroused by the sensation ; and the seat of this we regard as cerebral. The sensation itself, which was conveyed to the sensory ganglia, was not of a kind to excite the respondent motion, and passed on to the cerebrum without doing so ; but the idea excited there reacts upon the sensory ganglia, and produces in them the condition which was excited in the former case by the sensation, and thus calls forth the respondent motion. And the same results will ensue, when the idea has been long stored away in the mind, and recurs involuntarily, or is called up by an exertion of the will. In any such case the presence of the idea, instead of acting upwards upon the reasoning processes, operates downwards upon the body, and discharges itself (so to speak) in an involuntary or emotional action. The emotional states, when they do not thus immediately affect the muscular system, become the subjects of higher mental operations, and influence the determinations of the will, so as to be most important agents in the regulation of the voluntary movements. This double action of the emotions, which was long ago pointed out by Bishop Butler, and which has not been (in our opinion) sufficiently considered either by physiologists or metaphysicians, appears to us to find its best and simplest explanation in the two series of operations which we have attempted to develope,—the one involving only the very simplest cerebral action (the formation of ideas), which at once reacts downwards upon the body through the sensory ganglia, which give to those ideas the feelings connected with them,—whilst in the other, those ideas act upon the higher mental processes, and affect the body only through the will. And there is a well-known fact in our psychological and physiological history, of which this view seems to afford a particularly apposite explanation,—namely, the relief afforded to violent mental emotions, by giving free scope to the bodily manifestations of them. Every one knows the discomfort of suppressing a yawn, how much it adds to our fatigue, and thus makes us increasingly desire the cessation of the discourse to which we are listening. We know, too, how a ludicrous idea continues to haunt us, if we do not let it explode with a hearty "guffaw." And we believe that it is a matter of equally familiar experience on the other side of the Irish Sea, that, when the combative and destructive propensities are strongly excited, they commonly evaporate speedily with the free play of the shillelagh; being much more likely to produce an enduring resentment, which shall call the intellect into exercise for its gratification, if the desired fight be prevented. This we believe to be the true physiology of the relief which some irascible persons find in a hearty d——n, in a violent slamming of the door, or in any other bodily effort to which the emotional condition prompts. The depressing emotions, in like manner, are often worked off with a good fit of crying, sobbing, &c.

We have dwelt long upon these topics, because we are not aware that they have been anywhere handled in a manner at all satisfactory; and having thus endeavoured to limit the functions of the cerebrum to that which we have a fair ground, from anatomical, experimental, and metaphysical evidence, to believe it to perform, we shall briefly inquire how far these views are applicable to the phrenological system as at present held, and what modifications they tend to introduce into it. But it may be desirable to recapitulate briefly the positions upon which we have now dwelt.

1. That the sensory ganglia supply all the conditions requisite for the reception of sensations, in the higher animals as in the lower; and that there is a class of actions excitable through them by the direct influence of sensations; to these we give the name of consensual.

2. That the sensations which excite these actions, also excite the feelings of pleasure and pain, which have their seat in the same ganglia. These feelings may receive different designations, according to the nature of the objects towards which they are displayed. Thus, attachment and dislike, affection and rage, joy and sorrow, and many other simple and elementary feelings, are but modifications or phases of pleasure and pain, which receive their different designations according to the character of the objects which excite them, the ideas which they arouse, and the mode in which they are manifested.

3. That sensations, the simple feelings connected with them, and the consensual movements to which they prompt, make up the sum-total of those operations to which the term *instinctive* is properly applicable; that these take place through the instrumentality of the *sensory ganglia;* and that none of those higher operations which involve the formation of ideas, reasoning processes, and volitional determinations, can take place without a cerebrum.

4. That the cerebrum is the seat of the formation of *ideas*, or elementary notions originating from sensations, and of all those higher *intellectual operations*, of which those ideas form (as it were) the pabulum.

5. That the occurrence of *ideas* in the cerebrum may produce feelings of pleasure or pain in the sensory ganglia, analogous to those which are produced by *sensations*.

6. That the tendency to the recurrence of a certain class of ideas, constantly connected with feelings of pleasure or pain, constitutes what is known as an emotion, desire, or propensity; and that this is composite in its nature, involving the cerebrum for the formation of the ideas, and the sensory ganglia for the feelings with which they are associated.

7. That certain ideas, which thus strongly excite the feelings, may also produce motions through the instrumentality of the sensory ganglia and their nerves; that these movements are involuntary in their character, and are excited by emotional states in the same manner as they are by direct sensations; and that they consequently belong to the consensual group.

8. That intellectual operations *may* take place, in which the feelings do not participate (as, for example, in mathematical or scientific ratiocination); but that the motives which regulate our personal conduct are, in great part, derived from the feelings attached to particular ideas or classes of ideas. When the emotional states thus act, in affecting the further course of the mental operations, they have no immediate agency upon the body; their influence being exerted through the will.

9. That the exertion of the reasoning powers, and the final determination which, in its action upon the body or the mind, we call volition or will, operates solely through the instrumentality of the cerebrum.

10. That the cerebrum has probably no direct connexion, however, either with the sensory organs or with the muscular system; but that it depends upon the sensorial ganglia for the reception of sensations, and for the execution of voluntary movements: this execution being still guided by the sensations received through those ganglia, and the act of muscular contraction being dependent upon their continuance.

The first and chief point of collision between these views and the ordinary phrenological system, is that which relates to the localization of the passions, emotions, &c.; but it would not, we think, be found difficult to reconcile the two, so far as this question is concerned. For it may freely be admitted that there *are* such classes of ideas as those grouped together under the terms benevolence, combativeness, philoprogenitiveness, or destructiveness; although the pleasure attending the act of entertaining them which causes them to be habitually kept before the view of the mind, and thus gives them the character of propensities, be not seated in the cerebrum, but elsewhere. Now if we find reason to adopt the phrenological system as a whole, the only modification it would, require would be to regard the different divisions of the cerebrum, commonly termed *organs*, in the light of instruments for the formation of the several classes of *ideas*, instead of being the instruments of the emotions or propensities, taken as a whole, into which these ideas so largely enter. And we think that our view further harmonizes well with the fact, that the exercise of *every* faculty, perceptional or intellectual, as well as moral or animal, may be attended with pleasure or pain; and that we may put the intellectual faculties into the form of propensities, just as readily as we do the passions and emotions. For instance, there are individuals who feel a delight in following out a beautifully-connected train of scientific reasoning, which terminates in evolving a simple principle adapted to explain a great variety of complex phenomena,—or in recognizing an analogy between facts apparently remote, which becomes the key to generalization or induction,—fully as keen as that which another will experience in carrying into operation a plan dictated by enthusiastic benevolence, or which will fill the whole nature of a third, in devising some scheme from which he anticipates a large amount of personal benefit. The tendency to exercise the reasoning powers, or to search for these analogies, which result from the pleasure thus experienced, must be admitted to be as much a *propensity* as benevolence or acquisitiveness; and if it be admitted that the former is a complex state, involving the capability of a certain kind of intellectual operation, and the capability of gratification from the exercise of it, we can see no reason why the same method of analysis should not be applied to the latter. The different classes of ideas may still be called perceptional, intellectual, moral, and animal, according to the character of the objects to which they relate.

We have no *a priori* objection to make to the doctrine, that these different classes of ideas may be formed by the special instrumentality of different parts of the cerebrum. We freely admit, too, that there is a general correspondence between certain forms of cerebrum, arising from the relative development of its different portions, and certain leading diversities of character; which might not unfairly be regarded as indicating that these several divisions are the special instruments of particular groups of intellectual or moral faculties. We by no means desire to hold back from the inquiry, whether this inference is not a most legitimate one, and whether it may not be further carried out, by the subdivision of these regions amongst the elementary faculties, tendencies, &c., so as to be able to assign to each part of the surface of the cerebrum its special function, and, on the other hand, to point to the instrument of every one of the mental operations. That every act of mind, of whatever character, is accompanied by a *material change* in the nervous centres—the two being so necessarily linked together in our present state of existence as to be altogether inseparable—we most firmly believe; and there is no medium, in our apprehension, between the supposition, that the whole cerebrum acts together in every mental change, however simple, and the idea that different parts of it are appropriated to different elementary processes, and that they are called into distinct or conjoint action, according to the nature of the mental action that is going on. The latter view appears to us to derive support from various anatomical facts, especially from the frequency with which we are enabled to prove, that large ganglionic masses are made up of distinct parts, having really dissimilar functions; and from the gradual increase in the complexity of the cerebral structure, arising from the successive addition of new parts, which we encounter in tracing it from its first appearance at the bottom of the vertebrated scale, up to its highest form in man. Comparative anatomy thus seems to lend us important aid in establishing the fundamental principles, or axioms, of phrenological science; for if the cerebrum were an organ as homogeneous in *function,* as it appears to be in *structure,* we should expect that its higher development would consist only in an increase of *size,* instead of involving (as it manifestly does) an increase in the number of its lobes, the formation of convolutions, and a gradual increase in the number of the latter, together with analogous complications of internal arrangement. The doubts which we feel called upon to express, as left in our minds after a careful review of the whole subject, with the aid of Mr. Noble's replies to the various objections and criticisms quoted by him, relate, therefore, not to phrenology or cerebral physiology as a proper object of pursuit, or as a legitimate department of scientific inquiry, but to the reception of the present system, as one founded upon a correct induction of the phenomena which it should comprehend, and as expressing the whole, or nearly the whole, of the truth regarding the psychical nature and operations of man. These doubts we shall class under two heads, as they arise from physiological or from metaphysical considerations.

In the *first* place, the facts of comparative anatomy seem to be very far from coinciding with the *main features* of the system, in regard to the respective functions of the three lobes of the cerebrum; since, as we have already stated, the rudiment of the cerebrum which exists in fishes, and which is still more developed in reptiles and birds, is the representative of the *anterior* lobe alone of the human brain; the middle lobe being first developed in the lower mammalia; and the posterior being not merely restricted to the higher, but being more developed in man, relatively to the remainder of the mass, than it is in any other animal. Now, as upon the usual phrenological allocation, the lower or animal propensities are situated in the posterior lobe, their instrument would seem altogether undeveloped in the beings in which they apparently possess the greatest force; whilst they attain their most complete evolution in that species, which is distinguished by his power of keeping them in subjection. We have already shown that the usual phrenological explanation of this difficulty, confirmed as it is by the history of human embryological development, is altogether incompatible with facts. But we will offer another which may tend to remove the apparent discrepancy. We have already pointed out that we are not always to regard those *actions* of the lower animals, which correspond with our own, as indications of the existence of *propensities* in them corresponding to those from which they emanate in ourselves; these propensities being, in fact, the states intervening between the exciting sensations and the resulting will, and being compounded of ideas and feelings. Now in beings which are altogether destitute of a cerebrum, the actions supposed to proceed from the animal propensities must be really consensual in their nature; and it may be questioned whether they do not remain so, in those vertebrata which have no posterior lobe to their cerebrum; the propensities—that is, the *ideas* of the objects to which they respectively relate, and the *feelings* connected with the mental consciousness or contemplation of them—being really restricted to the higher mammalia, in which they are designed to work upon the intellectual powers and the will, to contrive the means for their gratification. On this hypothesis, we should expect to find the posterior lobe attaining by far its highest development in man; since, in the well-regulated mind, the animal tendencies never act otherwise than through the reason and will—that is, in the form of true propensities. And this *gradual* removal of the actions in question, first from the domain of reflex into that of consensual action, and then from the consensual or instinctive to the volitional group, is precisely conformable to that which we find in regard to other operations of the nervous system—the act of progression, for example. We trust that our views may find more favour among our phrenological readers, from their affording them a feasible method of extricating themselves from this very awkward dilemma, without abandoning any of their fundamental positions.

Secondly, in so far as comparative anatomy has been appealed to in support of the details of the present system of phrenology, the deductions drawn from it are entitled to but little weight, for the following reasons. A large part of these deductions was made prior to our present improved acquaintance with the functions of the nervous centres, and the real character of many of the actions of the lower animals was greatly misunderstood. In saying this we would not be thought to do injustice to Gall; to whom we are under great obligations for the advances which he made in comparative psychology, and in the comparative physiology of the nervous system. We are not unmindful of the fact that he was among the first, perhaps the very first, to advocate the doctrine that the ventral ganglionic cord of articulata is the analogue of the spinal cord of vertebrata. Nor do we forget how much he contributed to the establishment of these views, in regard to the combined action of the instincts and of the reasoning faculty among the lower animals, which are now current among well-judging psychologists. But it cannot be forgotten that the whole doctrine of reflex action, and of the dependence of the greater part of the actions of invertebrata, with a considerable proportion of those of vertebrata, upon the spinal cord alone, has been established since his time; that the identification of the cephalic ganglia of invertebrata with the sensory ganglia of vertebrata has only been recently accomplished; that the reference of a large class of movements to these bodies, independently of the cerebrum, being consequent upon that identification, is only now claiming attention as a physiological truth; that the actions of which the cerebrum is the instrument have, consequently, not yet been clearly distinguished from those in which it has no direct participation; and that analogous *actions* may be referrible to entirely different *springs of action*, when we contrast one species with another, as when we compare the motives which operate in different individuals of the human race; so that we are not to infer the existence of a particular psychical propensity from the observation of a series of habitual actions, which may be really consensual. Further, in reference to the mere anatomical comparison, we must remark that enough pains has not been taken to bring together facts which are really similar. We hold all comparison of *crania of different species* of animals to be utterly worthless as furnishing materials for phrenological deductions. It affords no key whatever to the comparative development of different portions of the cerebrum, as is shown by the fact that *similar* projections on the cranium, even in closely-allied species, are found to have under them *different* convolutions of the hemispheres; and by the total inability of this kind of examination to lead us to an exact knowledge of the relative development of even the different lobes of the cerebrum, as is evident from the circumstance that there is no external distinction between the middle and posterior lobes, and that even the distinction between the anterior and middle may not be apparent in the cranium. Again, the external contour of the cerebral hemispheres, in all cases in which they bear a small proportion to the masses beneath them, will be greatly influenced by the form and size of those masses—a circumstance which has been totally overlooked in comparative measurements of the cerebra of different animals, both by phrenologists and their opponents. Moreover, we believe that the general form of the cranium, in different species of animals, is greatly modified by its instrumentality in other functions, especially in mastication, and by its position upon the trunk and mode of muscular connexion with it; and that the disposition of the several component parts of the encephalon may vary with the general form of the cranial cavity, so that no valid inferences can be drawn from the latter as to their relative size. All observations made upon crania of different animals, therefore, ought, we think, to be put aside; and those data alone to be received which are derived from careful examination of the cerebrum itself. And with respect to nearly all the recorded observations upon the conformation of the hemispheres, we may remark that they are open to objection, on the ground of a want of sufficient acquaintance with their anatomical structure, to warrant us in receiving all those phenomena as really analogous which profess to be so. The only true rule—the applicability of which to the nervous system becomes more apparent, the more we become acquainted with the fundamental nature of its operations—is to compare *like* things with *like*. Now the cerebrum of a bird is *not* like that of a mammal as a whole, but can be compared only to its anterior lobe; and it seems absurd, therefore, to be looking there for the organs of combativeness, philoprogenitiveness, destructiveness, or secretiveness, which belong to the posterior and middle lobes. What, then, becomes of all such organs? We reply that it is much more easy to conceive of their having no existence in the bird's mind as propensities, the actions referred to them being simply consensual, than it is to suppose that the uniformity which we everywhere see in nature is so egregiously violated. The only safe guide in this department of comparative anatomy seems to us to be to follow out the divisions which nature indicates to us—namely, the cerebral convolutions—in all animals in which they exist; and to devote our first, or even (for a time) our sole, attention to these; at the same time, endeavouring to analyse the respective characters of the different animals, with especial regard to the objects upon which their intellectual faculties are employed, so as to distinguish between the merely consensual actions and those which result from the operation of the will guided by psychical propensities. Until all the knowledge has been acquired that this kind of concurrent investigation is capable of yielding, we think it altogether unprofitable to attempt to investigate the cerebral physiology of animals on whose hemispheres there are no convolutions. We shall not stop to discuss here how far the respective observations of Gall, Vimont, Tiedemann, Leuret, and others, are valuable for the end we have in view; our conviction being, for the reasons already stated, that the whole subject must be investigated anew; and the figures and descriptions of all the authors we have referred to being far from sufficient, in our estimation, as the foundation of such an investigation.

Thirdly. We cannot feel at all satisfied, that the method of determination hitherto employed, as regards the human cerebrum alone and its functions is sufficiently trustworthy. The greater part of the observations upon which the details of the present system of phrenology rest have been made upon crania alone, or upon casts, which are still more fallacious. Now although we do not charge this method of observation with being as open to objection, in comparing different individuals of the same species, as it is in comparing different species with each other, yet still we think, that its results are likely to be erroneous and imperfect; for the following reasons. There appear to be, in different races of men, *typical forms* of skull, the general contour having little or no relation with the comparative development of different parts of the encephalic contents, but simply modifying their disposition in the cranial cavity. This assertion we make upon the authority of Professor Retzius, who has studied this department of ethnology more profoundly than even Dr. Prichard himself, and who has shown that the classification of nations, formed according to the shape of the cranium, has so little correspondence with that which would be based upon their correspondence or diversity of psychical characters, as to indicate that no inferences can be drawn from one class of facts in regard to the other. We do not think that phrenologists can hesitate in admitting the possibility of two brains, possessing the same relative development of their respective parts, being moulded into two different shapes, according to the form of cranium peculiar to each race; since they have always admitted the possibility of much more extensive displacements as results of disease. But further—and this is, perhaps, our most serious class of objections to the *present system* of phrenology—no external examination of the cranium, such as can alone be made on the living head or the unopened skull, can give any account of the form of the *base* of the hemispheres, which, being covered with convolutions equally with any other part, must be supposed to have a like participation in the cerebral functions. And even when the examination is made upon the cerebrum itself, the large surface of each hemisphere in apposition with the falx is altogether neglected. Further, as the inner surface of each hemisphere is always flattened against its fellow, no increased development of any part of it can manifest itself, except by thrusting out a corresponding portion of the external side of the hemispheres; so that a protuberance of a certain portion of the latter may be due to a cause altogether irrespective of increased development of the organ to which it seems to belong. Now from the difficulty of determining the development of the basal portion of the cerebrum, and from the absolute impossibility (in the present state of our anatomical knowledge) of judging, even from the cerebrum itself, of the development of the internal portion of each hemisphere, it has happened that scarcely any attempt has been made to map out the *base* of the brain into organs, and no attempt whatever has been made to show what share is taken by the internal surface of the hemispheres in the psychical operations of man or any other animal; so that we are scarcely beyond the mark in asserting that nearly (if not quite) *one half* of the cerebral surface is *totally unappropriated.* Yet not the slightest ground can be adduced for the supposition that these unappropriated portions have any less participation in the operations of the intellect, the exercise of the moral feelings, or the influence of the animal propensities, than have the external and superior portions of the respective lobes. We have met with some phrenologists who were candid enough to admit this to be a difficulty of which they could not give any explanation; their belief being that further observation would lead to increased knowledge of the subject. But in no system of phrenology that we have consulted have we met with an admission on this point, at all adequate to the extent of the difficulty; for, be it observed, one of the great claims which is set up in behalf of the phrenological system is the completeness of the system of psychical philosophy which it presents; the whole series of mental phenomena being arranged under certain categories, each of which has its corresponding representative on the cerebrum. Now if this be the real state of the subject, what are we to believe as to the geography of the terra incognita not yet explored by phrenologists? Are there no "organs" in it? If there be, for what sort are we to look? And if there be not, how can their absence be explained?

The fourth and last of our sources of doubt, in regard to the physiology of the present system of phrenology, arises from the want of sufficient accuracy in those observations upon the human subject, upon which it professes to be founded; and from the prevalence of the habit of attending to and recording the *coincidences* between certain cerebral developments and psychical manifestations, without due regard to the cases in which there is *no* coincidence. The difficulty of precisely recognizing the relative sizes of different organs from external examination of the head has often been acknowledged and regretted by phrenologists themselves. It leads to the formation of very different estimates of character from the same data, as we can testify from our personal experience; three extremely diverse accounts having been given of our own "developments," by three well-known phrenologists, in the course of a few months. In another case, which happened to no less an authority than Spurzheim, two very different characters were given of the same individual (a young lady) within half an hour, under the following remarkable circumstances. Dr. Spurzheim was requested to examine the heads of two young ladies, sisters, who so closely resembled each other in person that the wearing of a cap by one of them was necessary to enable even their parents to distinguish them, and who yet differed considerably in psychical character. The capless young lady having undergone the doctor's manipulation, left the room, put on her sister's cap, and returned for a second scrutiny, which was made under the impression that the other sister had appeared; and an extremely different statement of her character was then given. Now either Dr. Spurzheim *intentionally* varied his account, to meet what he believed to be the difference in character between the sisters; or he was unconsciously influenced by the expectation of finding a difference in the developments, the want of any accurate method of measurement causing him to make an involuntary error in his estimate. Our readers may adopt which explanation they please; but the fact, which we have heard upon the most excellent authority, indicates (however explained) that the absence of such a method, by which fallacies of either kind could be detected, invalidates all the observations of Dr. Spurzheim, and leads us to the suspicion that, in other cases also, the process of taking the developments is open to serious error. And further, there is an equal, if not a greater difficulty, in a large proportion of cases, in the acquirement of such a correct knowledge of the character of the individual, and of the *hidden springs* of his actions, (which are often very different from those which would be supposed to be in operation, even by his most intimate relatives and friends,) as shall enable us to ascertain with certainty whether a coincidence exists or not. Every one knows how readily, in phrenological examinations, as in mesmeric performances, such coincidences are caught at; and we are satisfied, from instances which have fallen under our own observation, that a certain coincidence of the *conduct* with the line marked out by phrenological prediction, may result from a *character* very different from that, which the cranium was supposed to indicate, and which gave no indication of actions and feelings known only to the individual himself. All observation, then, which has reference to points of character, about which there can be any doubt or mistake, is valueless for establishing any organological system; and the only valid indications are to be derived from the observation of individuals, in whom the manifestation, or the contrary, of particular faculties or propensities is so decided, that there can be no reasonable hesitation as to their existence to an unusual degree, or their deficiency. We must do Gall the justice to remark, that this was the method adopted by him in his investigations; and if his followers had restricted themselves to the same practice, until a determination of organs had been thus effected, which should be generally satisfactory to phrenologists and metaphysicians, we believe that phrenology would be at present in a state of more real, though less apparent advancement.

But all observations, however carefully made, however sagaciously interpreted, are utterly valueless, unless the failures are chronicled equally with the successes. Will any professed phrenologist, of known candour, come forwards, and say that he has done so, and that he has given his *entire* experience to the public? We do not address our query to those whose minds are so engrossed by the spirit of system, as to consider their science so perfect and unassailable, that no objections *could* be brought against it, which they would be unable to explain away. Nor do we desire an answer from those who have long considered the propagation of phrenology the sole business of their lives, and whose notoriety entirely depends upon their advocacy of it. But we put the question, in thorough good feeling, to those who have become persuaded of the truth of the present phrenological system, after what they deem a sufficient course of inquiry; who can say in singleness of heart that they have no personal motive for upholding it; and who are competent, from their habits of observation, from their knowledge of human character, and from their acquaintance with their own mental constitution, to judge when there is a real coincidence and when there is not. We lay great stress upon this last qualification. We all know more or less from our own experience the truth of the saying "we easily believe what we wish," and unless we carefully watch ourselves we are continually being influenced, in our scientific observations and deductions, by our feelings regarding the question upon which these bear. This influence may be exerted quite unconsciously, and therefore innocently, on the part of the individual himself, though it is frequently obvious enough to others; and we ought, therefore, to be doubly on our guard against it. We think we perceive many traces of it, for instance, in the present work; though no man is more capable than our author of pointing out a similar fallacy in the pretensions of the mesmeric system, or of detecting the sophistry that lurks in all accounts of wonderful cures resulting from the employment of a particular remedy or method of treatment. We believe that phrenologists, like mesmerists, and too many doctors, are continually subject to this kind of influence, which has the more scope for its exercise, the greater is the difficulty of making an *exact* system of observations. The chemist who can weigh his reagents with the greatest nicety, or the astronomer who can measure the movements of the heavenly bodies to a marvellous degree of accuracy, is far less subject to errors of this class; yet neither is exempt from it in the process of forming deductions from such observations, as a survey of the history and present state of chemical and physical science would most abundantly show. In phrenology this source of error is liable to operate alike in cranioscopical observations, in the process of deduction from it as to the probable character, and in the estimation of the real character; and it needs but a very little aquaintance with the doctrine of chances to be aware that the introduction of even a slight element of uncertainty into three distinct processes bearing upon one another, most seriously impairs the probability of correctness in the final result. We could not, therefore, by any means trust to the coincidences, even if honestly recorded with *all* the failures; unless we knew the character and qualifications of the party by whom the observations were made. And our present estimate of the phrenological mind is not sufficiently favorable to lead us to accept the published records of the coincidences as a fair expression of the entire evidence on the subject. For example, we know of an instance in which Dr. Spurzheim pronounced the organ of number to be *deficient* in an individual who was at that time known as the "calculating boy," and who is now an engineer distinguished for his readiness at computation; and we have known the *absence* of the organ of colour to be stated by an eminent London phrenologist as the only remarkable point about the head of a man who was possessed of such powers as a modeller as to be able to produce an exact coloured representation, by the aid of memory alone, of any object to which his attention had been directed. We have heard of many such failures, but they do not find any record in phrenological publications. And the reason why they are not duly chronicled by the opponents of the system, and set in array against the imposing results of Mr. A.'s visit to a prison, Dr. B.'s to a madhouse, and Mr. C.'s to a school or factory, is simply because no one has thought it worth while to accompany Mr. A., Dr. B., and Mr. C., upon their various perambulations, and to note the results throughout. We do not mean to assert that the failures *would be* so numerous as to invalidate the positive results, when both are fairly collected and compared; but we do assert that, *until* such a collation has been made, no more weight is to be attached to the records of positive or coincident observations, than we should admit to the marvels of clairvoyance, or to the accounts of the cure of consumption by a particular remedy, both which are detailed to us by numerous witnesses, who would not knowingly propagate an untruth. There is one more fallacy, which we would point out as resulting from the want of sufficiently accurate means of measuring craniological developments, and from the facility with which the mind of the observer is influenced by extraneous considerations; and this is, the information conveyed by the expression of countenance and general deportment, which, even when not a word is spoken, is often sufficient to afford a fair general knowledge of the character of the individual. We believe that many of the collections of observations, which seem to indicate the triumphant success of the present system of phrenology, especially those made in prisons and lunatic asylums, might be made with nearly, if not quite, equal success, by a good physiognomist; and until a fresh and impartial collection of such observations shall have been made, in which this source of fallacy shall have been excluded by the interposition of a mask, or by some similar means, we cannot feel that they ought to be relied upon as contributing, in any important degree, to the support of the existing phrenological system.

It is against *this system,* and not against phrenology, or cerebral physiology as a legitimate subject of investigation, that we have felt called upon to adduce the foregoing objections, derived from the want of correspondence between its dogmas and the unquestionable facts afforded by comparative anatomy, and from the fallacious and insufficient character of the evidence on which it mainly rests. And we believe that it will share the fate of other systems, religious, ethical, and physical, which have been upreared with similar haste and upon a like insecure foundation; being destined, if not to be undermined and overthrown by more persevering and well-directed inquiry, at any rate to be reconstructed on a plan which shall embrace wider foundations, and be followed up with more securely-laid courses. It is no disgrace to the early framers of the system that it should now require such extensive modification; for the whole science of physiology has been remodelled within the same period, and the almost utter ignorance which prevailed when they pursued their labours, in regard to the special functions of the nervous system, has been in great part cleared away. That the Gall-and-Spurzheim system of phrenology should require, therefore, something more than the slight additions and changes it has undergone at the hands of Mr. Combe and his coadjutors, to reconcile it with the present comparatively advanced state of our knowledge of comparative anatomy and of general neurology, cannot be deemed a bold assertion. Nor should those who volunteer their aid in this reconstruction,—by bringing as materials their stores of knowledge, acquired by modes of investigation totally neglected by the professed followers of Gall and Spurzheim, and by offering to unite their labours with those of the latter, if they can only be assured that a fair use will be made of their work, and that it will not be thrust aside for contributions of really inferior value, but presenting a more specious appearance,—be treated as opponents and destructives. We have endeavoured to earn for ourselves the credit of directing the public mind in the estimation of the present claims of mesmerism; and more recently we have ventured to question the time-honoured systems which at present constitute what is known as medical science. In both cases we have endeavoured to separate the specious from the real, to show how one doctrine after another, that at first commands the general assent of those to whom it is presented, is scattered to the winds when its pretensions are fully and fairly analysed, (a few grains of corn being perhaps left, after a whole bushel of chaff has been thus dissipated), and to indicate the methods which will be more likely, in our estimation, to evolve solid and substantial truth. We have been endeavouring to do as much for phrenology, and would claim, on that account, to be regarded as the real friends of the science *as it may be*, in spite of the opposition of the professed advocates of the *science as it is*, for which we are fully prepared.

In order that we may put our readers in possession, so far as possible, of the whole state of the question, we must ask their attention to certain metaphysical difficulties, or rather deficiencies, attendant on the present system of phrenology. We should not think it necessary to point out these, were it not for the claims which phrenologists have set up, as the expounders of a new system of psychical philosophy, far more perfect than any which had been offered to the world from any other quarter; capable of explaining all the diversities and contrarieties of human character, and therefore better adapted than any system that can be formed independently of phrenology, to be the guide in education, in the treatment of criminals, and in many other momentous decisions which deeply involves the welfare of the human race. Such claims can only be set up by persons of very shallow acquaintance with the conclusions formed by intelligent observers of human character (to say nothing of professed metaphysicians, who are not always the best guides in such matters), antecedently to phrenology, or independently of it. We have known an individual, for example, who had been practically and most successfully engaged in education for upwards of forty years, assured by a leading professor of the phrenological system of education, that the "new philosophy" would afford him vast assistance in his vocation; but after sedulously attending the course of lectures, in which this system was expounded, our informant declared that he had not heard a single principle enunciated which had not been constantly in his view, from a time when the claims of phrenology were unknown in this country. We believe that it would not be difficult to show, too, that the advances made within the last thirty years towards a better understanding of the nature of mental derangement, and the improvements in the treatment of that condition, are due fully as much to the general progress of inquiry upon these subjects, as they are to purely phrenological investigation. But we are quite ready to admit, on the other hand, that the writings of phrenologists have done much to accelerate that progress, especially by fixing attention upon the *propensities* as the chief springs of human action, and by bringing into prominent view the fundamental diversities in the characters of different individuals, resulting from original differences in the relative force of those propensities. And we are quite ready to give full credit to Gall, as the first (we believe) to enunciate clearly the true relations between the psychological character of man and that of the lower animals, by pointing out that, in the former, as in the latter, instincts are at work, as incitements to action; and that, in many of the latter, as in the former, these instincts are placed under the guidance of reasoning powers, more or less developed. But all these views may be and are entertained quite independently of the organology of phrenology, and cannot therefore be said to be in any way peculiar to the upholders of that science.

With regard to the merits and deficiencies of the purely psychological portion of the phrenological system, we cannot here enter upon them at any length. For a very able, though brief, examination of them, we may refer our readers to a valuable and impartial work,* lately published by a gentleman, whose extensive acquaintance with the existing systems of psychology, and whose unprejudiced judgment, render him peculiarly capable of pronouncing an opinion on this topic. That he is by no means insensible to the benefits which science has derived from phrenological investigation, appears from the following conclusion to his preliminary remarks upon the subject: "It has, in a word, thrown a light upon our knowledge generally as to the functions of the encephalon, which did not exist before, and so far has conferred a benefit upon the science of man, which it were uncandid not to acknowledge." "But with these, its physiological researches," he continues, "as it appears to us, the whole of its advantages terminate." It is clearly shown by the author to whom we refer, that no organological comparison can suffice to evolve a system of psychology. "If every organ had its name and nature inscribed upon it by the Creator, then we should have a system of psychology at once; but, so long as this is not the case, we must observe and classify our mental phenomena by reflection before we can begin to map out the locality in which they are to be found." "Strictly speaking," he continues, "phrenology cannot reveal a single intellectual fact which was not equally known before; it cannot trace any points of human knowledge to their primary elements; it cannot perform in any case a single analysis of our complex notions; in a word, it can do nothing, allowing its facts to be true, but point out a certain connexion between two parallel series of mental and physical phenomena." If it be urged that the very circumstance of different feelings or faculties operating in connexion with certain portions of the brain is a clue to a correct classification of them, it may be fairly replied, with Mr. Morell, that "we did not require any phrenological aid to convince us that the animal passions, the moral feelings, and the intellect, present three different classes of phenomena, which cannot be perfectly resolved into each other." And in regard to the more detailed allocation, if we take into account the uncertainty and indefiniteness of phrenological investigation, and the admitted sources of error attending its determinations,

* History of Modern Philosophy. By the Rev. J. D. Morell, A.M. 1846.

even if the fundamental principles on which they rest are conceded, we may well doubt "whether the slightest aid could ever be afforded by phrenology in analysing our mental phenomena;" nor can we yet believe "that a classification grounded upon the position of the organs can be in any way so satisfactory as one which is grounded upon an accurate observation of the mental phenomena themselves." In fine, with regard to some of the most important problems of metaphysics and morals, phrenology has never attempted a solution, and cannot afford the slightest aid in the search for it. "The only thing it attempts is to ridicule the questions themselves; which is a method of treating them alike easy and ignoble." After glancing at some of these questions, Mr. Morell thus concludes: "We repeat, therefore, that phrenology ought to have taken its place as one branch of physiological investigation; that, viewed in such a character, it has succeeded in educing many interesting and valuable facts respecting the material changes which accompany the exercise of thought and feeling; but that, in attempting to take its stand as a system of intellectual philosophy, it has entirely mistaken its proper place, and totally failed in throwing any light whatever upon moral or metaphysical researches."*

Only one portion of the encephalic mass now remains for our consideration, namely, the cerebellum. The anatomical distinctness of this from the other nervous centres is so marked that no physiologist entertains a doubt as to its speciality of function. With regard to the general nature of that function, there is an almost universal agreement amongst those physiologists who attach most weight to comparative anatomy and to experiment; whilst, on the other hand, an entirely different function is attributed to it by those who place the greatest confidence in observations upon man alone. In regard to the nature of the evidence supplied by comparative anatomy, we shall here only observe *in limine* that it affords us the means of readily contrasting the extreme states of development of the organ within the limits of the vertebrated series; for we may trace its gradual diminution, as we descend the scale, through the higher mammalia, the lower mammalia, birds, reptiles, and fishes, until we lose it altogether in the lowest of the latter class. Such a comparison we must regard as more likely to evolve the truth in regard to its function than any collection of observations upon the human cerebellum, in which the size and weight of the organ have not been most rigorously determined. Mere craniological observations we must regard as totally valueless, at any rate until a much more complete knowledge has been attained as to the influence of particular types of conformation of the skull; for the observations of Professor Retzius upon the varieties of form which the cranium presents in different races have indicated this among other facts,—that the *position* of the cerebellum may vary considerably, being much more horizontal in one case and more vertical in another, so as to correspond with a greater or less posterior protuberance, without any corresponding variation in the size of the organ itself. And we shall presently see that Gall was misled by his craniological observations, in estimating the influence of castration upon the condition of the cerebellum.

* Op. cit., vol. i, pp. 411-26.

In regard to experiment, again, this organ affords peculiar facilities. It can be removed, partially or completely, without any very severe lesion of the remainder of the encephalon; and animals may long survive the operation, and indicate the results of the ablation of the organ, after the immediate effects of the injury have passed off. We do not hesitate to affirm that there is as great an agreement between the actual *results* of experiment upon the cerebellum, as there is in regard to those of any other class of physiological experiments, involving numerous precautions, and attended with peculiar sources of fallacy; although the *inferences* deduced from these results have varied, according to the preconceived notions of the respective experimenters. Of these variations the closest followers of Gall have not been slow to avail themselves, by way of excuse for putting aside the experiments altogether, as totally valueless for yielding any results that can be at all depended on; yet there have been not a few of the upholders of his general system, who have admitted the force of the evidence, and have adopted those deductions from it which are now received by most physiologists, without finding it necessary to give up the doctrine of Gall regarding the connexion of this organ with sexual desire. Mr. Noble, however, is not one of these; and we must own ourselves completely at a loss to understand how his usual acuteness of perception should have so unaccountably deserted him, as it seems to us to have done, in the analysis and contrast of the experiments of Rolando, Flourens, Magendie, and others, into which he enters near the commencement of his book. The only solution, in fact, that we can offer of his want of perception of the fundamental accordance of *all* these experiments, is that he has been so blinded by his preconceived views, as to be able to perceive only the points of difference, which exist, as we shall presently show, rather in the minds of the several interpreters of the experiments than in the results themselves. Thus he says:

"It will thus be seen that no two of the above instances presented anything like coincidence in the results; but that, on the contrary, direct contradictions occur. Rolando's paralysis is met by Bouillaud's no paralysis; Flourens' *inability* to *regulate* movement is counterpoised by Magendie's *capability*, confirmed by Fodera's experience; and the same contradiction is seen throughout the entire history of these vivisections. There is no single fact recorded by one operator which is not counteracted in its tendency to any conclusion by the experience of some of the others." (p. 25.)

The ordinary physiological view of the functions of the cerebellum, founded upon the experiments above referred to, and confirmed by numerous others, as those of Hertwig, Budge, and Longet, which Mr. Noble does *not* cite, is that the cerebellum is the organ for combining and regulating *voluntary* muscular actions, in which the united action of many muscles is required; especially those concerned in locomotion, and in the maintenance of the equilibrium of the body. This harmonizes well with the fact, that *no cerebellum exists where there is no cerebrum;* and that there is a pretty general conformity between the relative development of the two organs. Where the movements are purely *consensual,* still less when they are *reflex,* no cerebellum can be required to regulate them; and we therefore are surprised that Mr. Noble should have quoted (p. 255,) the regular movements executed by the decapitated trunks of insects, as having any bearing on the question. He surely does not mean to affirm, that in a man, whose spinal cord has been divided in the dorsal region, and whose limbs execute the most powerful reflex movements, any kind of stimulation will cause these limbs to execute the combined movements requisite for the maintenance of his body in the erect position, still less, to perform the act of progression. If this cannot be effected, the balancing and co-ordinating power must have its seat in the encephalon of those animals, whose movements are not purely reflex. We see abundant proof that, in the invertebrata, the encephalic power over the movements of the body is limited to the control and direction of the actions, which, in themselves, are individually reflex; thus, in one of Mr. Newport's experiments upon a centipede, the removal of the ventral cord from the middle of the trunk caused the complete paralysis of the members immediately connected with the abstracted ganglia, whilst the posterior members continued to move and to force the body onwards, in harmony with the members anterior to the part of the cord removed, so long as the latter were in action; but when the former *ceased,* in consequence of an obstacle placed before the animal, of which it evidently took cognizance through the medium of its eyes, the latter *continued,* in consequence of their removal from the control of the cephalic ganglia. We have no reason to believe, then, that the purely *consensual* actions, however complicated, require such an instrument for the combination of the muscular movements needed to execute them; since the complete absence of the cerebellum in invertebrata shows that all which the sensory ganglia effect may be accomplished without it. We may, therefore, infer with certainty, that all the consensual movements in man and the higher animals,—such as sneezing, laughing, vomiting, and the like—are independent of this organ, the muscles being called into combined action by the direct influence of the sensations. But a little consideration will show, that movements executed in obedience to the will, of which the cerebrum is the instrument, are likely to need some such special organ for their regulation; for the will seldom exerts itself *directly* upon the muscular system, if, indeed, it ever does so at all, —its power being restricted to the issuing a general mandate (so to speak) for the execution of a certain action, of which the intellect has conceived the idea; and the selection and co-ordination of the various instruments, by which that action is to be accomplished, being effected altogether without any power on our own parts to direct it. We doubt whether we can will to put *any* muscle into action, independently of the rest; our power being limited to the execution of a particular movement, in which we know that muscle to participate, but in which its action is accompanied by that of others, from which we cannot isolate it. The only cases in which we *appear* to have such a power over individual muscles, are those in which the movements are so simple as to require only the single action of those muscles for their performance.

It appears to us perfectly conformable, therefore, with our general knowledge of the relative operations of the encephalic centres, to suppose that an organ should exist for co-ordinating the movements of the different muscles, in the various actions, most of them possessing a considerable degree of complexity, which are performed by the centres of the motor system of nerves, in respondence to the mandates of the will. And the anatomical connexions of the cerebellum with the sensory ganglia, through which the sensations are conveyed that *guide* the individual movements, and with the medulla oblongata, through which the motor impulses are conveyed to the nerves, harmonize well with such a supposition. The peculiar connexion of the cerebellum with the sensory ganglia was supposed by Foville to indicate that it is the actual seat of sensation; an idea at once negatived by the entire absence of the organ in invertebrated animals. We shall now proceed to inquire how far the above doctrine is borne out by the results of experiment, and how far the discrepancies pointed out by Mr. Noble should lead us to reject those results as valueless. We would first remark, however, that we must carefully bear in mind the difference between the results of the simple ablation of the organ, and the direct results of the injury which that ablation involves. All convulsive movements fall under the latter category; for, as will presently be shown, they subside as the shock and irritation pass off; whilst we are to look in the residual phenomena for the simple results of the loss of the organ. How much may be learned from the latter, after making all necessary allowance for the former, will appear, we think, from the following analysis.

In the experiments of Rolando, made in the infancy of our knowledge of the physiology of the nervous centres, the severe injury or removal of the cerebellum caused inability on the part of the animal to sustain itself upon its legs, or to execute any movements of progression; the animals experimented on soon died, however, with convulsive spasms and hemorrhage. Rolando was *previously* possessed with the view, that the cerebellum is the sole source of the muscular movements, and he represents the ablation of the cerebellum as producing hemiplegia or complete paralysis (according as one or both hemispheres were removed), disregarding the fact which he records, that movements both of the anterior and posterior extremities, *not* of a convulsive nature, were observed after the operation. These movements he attributed to the mere mobility of the muscular fibre, or to some remnant of the cerebellum! We may safely affirm, then, that these results are in no respect contrary to those of Flourens and others, in which no paralysis was indicated. Between the results of the experiments of Flourens, Bouillaud, Budge, and Longet, there is a complete coincidence in regard to the essential fact, that a mammal or bird, deprived of its cerebellum, cannot maintain its equilibrium or execute any regular movements of progression, although it can still move its limbs with energy in all directions. And parallel experiments upon the removal of the cerebral hemispheres fully prove, that the severity of the operation is itself not the cause of the result, since this much more severe operation does not produce it, animals being able to stand, walk, fly, &c., as well after the removal of the cerebrum as when it is entire. "Take two pigeons," says Longet, "and remove from one of them the whole of the cerebral hemispheres, and from the other only one lobe of the cerebellum; the next day the first will be firm upon his feet, whilst the second will exhibit the unsteady and uncertain gait of drunkenness." But, it is urged by Mr. Noble and other adherents of Gall's views, all these results are in direct opposition to those of Magendie and Fodera; in which the effects of the removal of the cerebellum appeared to be confined to the imparting of a tendency to a peculiar *direction* of movement, the power of executing combined movements being asserted still to exist. Magendie found that division of one of the crura cerebelli caused the animal to continue rolling over towards the corresponding side, and that deep wounds of the organ occasioned a tendency to retrograde movement, even when the animal made an effort to advance. These disorders may continue for many days, or even weeks. In the experiments of Fodera, there was an evident disturbance of the motor function, but no constant result. The animals seem to have all died from hemorrhage within a very short period after the operation. Now, these results are readily explicable on the idea, that the sensory ganglia are the proper centres of all movement which is not reflex, for we find that any disturbance in the condition of the latter, or of the *guiding* nerves connected with them, may produce the same result. We have already referred to the tendency to move in particular directions, as a consequence of section of the nerves of the semicircular canals, of the destruction of one eye, and even of a temporary obscuration of vision; the same result follows deep wounds reaching to these bodies; and it is not surprising, therefore, that lacerations of the commissural fibres connecting them with the cerebellum should produce a similar effect. Now that the irritation of these fibres is the cause of the peculiar movements in question, is shown by the experiments of Budge, who found that the movements would spontaneously subside after a time, but that they might be re-excited by irritating the cut surfaces of the crura cerebelli. With regard to the regular combined movements, said to have been observed by Magendie, we find none particularized but this,—that, on vinegar being held under the nose of a guinea-pig, whose cerebellum and cerebrum had been removed, it sneezed and rubbed its nose with its paws. These actions are evidently either consensual or purely reflex; and we might just as well quote the respiratory movements, or the efforts made by a decapitated frog to push away the probe that irritates its cloaca, or to grasp the female, as adduce the sneezing of a guinea-pig and the rubbing of its nose, when an irritating odour was applied, in proof of the power of co-ordinating the muscles for *voluntary* movements. In no record of experiments upon the cerebellum, save in those of Fodera, do we find that the capability of sustaining the equilibrium, or of executing combined and regular movements of progression, remained after the removal of the organ. The experiments of Fodera, as narrated by Gall, seem to have given but very imperfect results, partly in consequence of the very early death of the animals which were the subjects of them; and the narration is not sufficiently distinct to allow the real character of the movements to be made out. And as it is proved that the severity of the operation does not produce the result, we have no resource but to believe that the cerebellum is the organ directly concerned in the combination of muscular actions required to sustain the equilibrium or to produce these movements; or, on the other hand, to adopt Mr. Noble's hypothesis, that the admitted disturbance is a result of sympathetic affection of the motor apparatus. In regard to this hypothesis, we may remark, that it is partly founded on the imperfect discrimination, to which we have already alluded, between reflex and consensual movements on the one hand, and co-ordinate voluntary movements on the other; and partly also upon the want of due discrimination between the constant *loss* of power, consequent upon the removal of the organ, and the occasional, but not necessary, *disturbance* of the motor apparatus, which is equally occasioned, as we have seen, by other causes acting upon the ganglionic masses at the summit of the medulla oblongata. Between the two hypotheses, we think that the data supplied by comparative anatomy may assist us in forming a choice.

Before discussing these, however, we must make a few remarks upon the doctrine of Gall,—that the cerebellum is the seat of the sexual propensity. It seems to us that there are certain *a priori* objections to this, which have not been sufficiently considered, and which are not without weight, the uncertain character of the evidence in favour of the doctrine being a matter of notoriety. We readily admit that no *a priori* objections should stand in the face of anything like positive proof; but we cannot imagine that any candid phrenologist, who has duly considered *both* sides of the question, will affirm that such has been afforded. The first point we would urge is the evident *speciality* of the organ; not only as to its position and connexions, but also in regard to its internal structure. These seem to us to mark it out as an organ more distinct in function from the cerebrum, than the phrenological hypothesis would represent it, especially considering its very slight direct connexion with the hemispheres. For it cannot but be admitted, that the sexual propensity is as closely connected with many other propensities and desires,—e.g. with philoprogenitiveness, adhesiveness, and in the lower animals especially with combativeness,—as are any of the phrenological organs with each other; and that, in man, it has a continual operation upon the intelligence, the reasoning faculties, and the will. And yet we cannot point out any structural connexions by which this combination can be effected. Again, the size of the organ, and especially its amount of vesicular matter, in comparison with the cerebrum, looking at man only, is much greater than we can fairly expect it to possess, if we contrast the degree in which this propensity exerts its influence in the well-regulated mind, with the constant and energetic operation of others. Though its force is, doubtless, greater in some individuals, and its operation on the character and conduct more constant than external observers might suppose, yet in other instances, the propensity appears to be totally absent during the whole of life. Thus it is recorded of Newton, that he never gave the slightest indication of the existence of the sexual desire. And we believe that the early repression of it by a voluntary effort, as in the case of Catholic priests, disposes to its entire cessation for the remainder of life. Now there is an utter absence of proof as to any *strongly-marked* deficiency in the size of the cerebellum,—ascertained not by external observation of the skull, but by the dimensions and weight of the organ,—in cases of this kind; in which there ought, one would think, to be an almost entire absence of it, either congenitally, or by subsequent atrophy. We are continually told by the followers of Gall, that we should form our estimate from *extreme* cases only; and yet the most extreme cases on record, as to the size of the organ in man (as ascertained, we again repeat, by direct measurement), are not to be put in comparison with the difference in relative size that presents itself as we pass from man to the animals most nearly allied to him. To one other point we have yet to allude, namely, the early development and completeness of the cerebellum in the human embryo and infant; which is very remarkable when contrasted with the evolution of the cerebrum. We refer not merely to size, but to texture; the cerebellum acquiring the adult condition in this respect at a time when the cerebrum is still very soft and pulpy. This is precisely what we should expect, if its chief office be to regulate the movements of equilibrium, &c.; but is inexplicable if we suppose the cerebellum to be merely the organ of the sexual instinct.

We shall now take a brief review of the comparative anatomy of the cerebellum, with the view of determining to which of the preceding doctrines the data supplied by it give the most support. In the first place it is to be remarked that the organ is entirely absent in the invertebrata; yet sexual instincts are manifested most strongly in their actions. Here, as in the former case, we think a distinction must be drawn between the *instinct* and the *propensity;* the former being our expression for a certain series of phenomena directed towards a given purpose, but not really involving any other physiological or psychological actions than sensations and respondent movements; the latter being a desire for the gratification, involving an *idea* of the object. If we are right in this analysis, the sexual actions of insects, &c., may be purely consensual, and may not involve a *propensity* properly so called; and the absence of a cerebellum in them is not inconsistent with the phrenological doctrine. Neither is it opposed to that of Flourens and his followers, since all the movements of these animals are evidently of the consensual and reflex character.—The class of fishes may be looked to as furnishing important evidence; because, on the one hand, we meet with a greater diversity in the development of the organ than we encounter in any other class; whilst, on the other, there are equal diversities in regard to both classes of functions referred to it. The cerebellum presents itself in some fishes as a simple lamella, implanted transversely on the medulla oblongata, behind the optic lobes. In general, however, it is more extended longitudinally, and covers-in the fourth ventricle. Its highest development is attained in the cartilaginous fishes; in some of which it is marked by transverse furrows, dividing it into lamellæ more or less distinct, and thereby increasing its extent of surface. Now it is well known that in a large proportion of the class of fishes there is no sexual congress; the fertilization of the ova being accomplished simply by the diffusion of the seminal fluid through the water in the neighbourhood of the spot where the eggs are deposited—the two sexes being led by their instincts to frequent the same localities for the discharge of the contents of their genital organs. Our knowledge of the habits of fishes in this particular must be admitted to be very limited; but still there would seem a broad line of demarcation between the copulating and non-copulating fishes, which might be reasonably expected to be indicated by a difference of size or conformation in the cerebellum, if this were really the organ of the sexual feeling. Again, the diversities in the mode of locomotion of fishes are extremely great. It is to be remembered that the correspondence between the specific gravity of the water and that of the body of the animal, renders the support and the balancing of the body a matter of comparative facility in all circumstances; and the chief differences are to be looked for in the manner of propulsion, and in the facility of combining different movements for the change of direction, or for the attainment of any other special object. In the eels and other fishes of a similar shape, the vermiform character manifests itself as much in the movements as in the aspect of the body. The propulsion is almost entirely effected by the flexion of the entire trunk, the special organs being nearly undeveloped; and this flexion may well be regarded as being equally reflex in its character with the similar movements of articulated animals. But in fishes of higher orders the movements of the body become more and more complex, additional members are developed, and a much greater combination of different actions is required, in order to produce any desired result; whilst at the same time the variety of movements of which the animal is capable are very greatly augmented. This is especially the case with the most active predacious species, which are not satisfied with lying in wait for any unwary fishes that may pass near them, or with gulping down the small fry dispersed in the waters through which they move, but which pursue particular objects for vast distances, follow them with the greatest address, and adapt their movements most remarkably to the capture of their prey. There can be no hesitation, we think, in assigning to the shark tribe a pre-eminence in this respect.

Now on contrasting the size and development of the cerebellum in the copulating and non-copulating fishes respectively, there would seem, at first sight, to be something of that correspondence which might be expected on phrenological principles. For the sharks, which all copulate, possess the cerebellum in its most developed form; and the rays, which are nearly allied to them, and which also copulate, are also remarkable for the size of their cerebellum, which, though inferior to that of the sharks, is more developed than that of any other known fish. But here the correspondence ceases. The sharks and rays are by no means the only copulating fish; for a sexual congress takes place also in the chimæridæ, the syngnathidæ, the siluridæ, and in the blennies and eels. In regard to the development of the cerebellum in the greatest part of these, we have as yet no information; but it is well known that in the eels this organ is very small, being much below the average of the non-copulating fishes. Thus among the copulating fishes we find not only the highest but also a very low development of the cerebellum. Again, amongst the non-copulating fishes there are many which approach very closely in this particular to the highest group of the copulating; for whilst several of the rays are destitute of the lamellæ, which distinguish the cerebellum of the sharks, there are many of the non-copulating osseous fishes which have a cerebellum nearly (if not quite) equal in size and development to that of the rays, and far larger in proportion than that of the eels; this is the case, for instance, with the cod and the pike. These facts would seem to us decidedly unfavorable to the doctrine that the cerebellum is the organ of the sexual instinct. Let us now examine them under another point of view.

On comparing the development of the cerebellum in different species of fishes, with the activity and variety of the movements, a tolerably close degree of correspondence may be readily perceived. In the little amphioxus, which leads the life of a marine annelide, there is not a rudiment of this organ to be detected. In the lamprey and its allies, which are nearly as inert in their habits, attaching themselves to their prey by their suctorial mouths, and possessing but little power of active motion in search of it, we find the merest rudiment of a cerebellum. It is somewhat larger in the eels, which are fishes of considerable activity, though their movements are of a very simple character. In the cod and other actively swimming species, in which the impelling movements are still chiefly performed by the undulations of the body and tail, but in which the balancing and directing powers are rather given by the fins (as has been proved by experiment), we find the cerebellum attaining a much greater development; whilst in the sole, turbot, and other pleuronectidæ, which pass the greatest part of their lives with one side of their bodies in contact with the bottom of the sea, this organ is comparatively small. The highest development of the cerebellum, as already mentioned, is to be found in the sharks, a tribe pre-eminent for the vigour and variety of their movements; and in the much-dreaded white shark it far surpasses in comparative size and complexity of structure that of any reptile, nearly equalling that of many birds. It may be thought that the large size of this organ in the rays (which only yield to the sharks in this particular), is opposed to our inference, since these fish are not commonly reputed to possess much energy or variety of movement; but it is to be noticed that their progression through the water is accomplished in a different manner from that of most other fishes, and really involves a combination of movement which is very unusual in that class. The rays, in swimming, make very little use of the flexure of the body, which is the chief means of propulsion in the remainder of the class; the greater part of the vertebral column being unusually solidified, and even the tail serving merely as a rudder. Progression through the water is accomplished by means of the *combined* actions of the pectoral fins; and these are exerted so neatly and easily, that the fish appears to slide along without the slightest effort. "When a skate," says a competent observer, who had no purpose to serve in making the remark, "is swimming at its ease and undisturbed, its motion is peculiarly smooth and gliding, and puts one more in mind of a kite sailing along with motionless wings, than any other motion which we are accustomed to see."* The same writer, after contrasting the varied and facile movements of the rays with the limited and laborious wrigglings of the flounder tribe, thus concludes: "We have been somewhat particular in describing the motions and organs of motion of these animals; because, on account of the peculiarity of their forms, there is a very general notion among persons not acquainted with the economy of the sea and its inhabitants, that they are very unwieldy and helpless creatures in the water; whereas the fact is quite the reverse, and, even among those fishes, which are regarded as models of perfection as swimming animals, there are none who have more command, or even as much command, of themselves as the skate." This testimony from an unprejudiced observer proves that the high development of the cerebellum in the ray tribe most completely accords with the character of their locomotive powers, instead of being opposed to it, as seemed at first sight to be the case.

The cerebellum of reptiles is, on the whole, smaller in proportion than that of fish. In no instance does it surpass in development that of the higher cartilaginous fish; and in general it is below the average of that of the osseous fishes. Thus in the frog it is a mere transverse lamina, not covering-in the fourth ventricle, and scarcely more developed than the cerebellum of the suctorial fishes. In the lizards and serpents it is but little larger in proportion; and it is only in the crocodiles and turtles that

* Mudie, in Cyclopædia of Natural History, vol. iii, p. 514.

it presents any considerable dimensions. It is well known that all reptiles have a sexual congress: though it is not always that there is an actual intromission of the male organ. Like most other acts in this remarkable class, it is very leisurely performed; often lasting several days, seldom less than one. The strength of the sexual instinct in these animals would seem to be indicated by the remarkable *preludes* which many of them perform; and by the battles of gallantry which are common among others. The frog has often been quoted by anti-phrenologists as an example of an animal possessing a strong sexual instinct, with a rudimentary cerebellum; its sexual instinct being supposed to be indicated by the duration of its copulation, and by the perseverance with which it continues to grasp the female, even after decapitation. But Mr. Noble urges (p. 330), and we think with justice, that this powerful and continuous prehension, being a purely *reflex* act, and excited (as Messrs. Todd and Bowman have shown) by the peculiar impressibility of a papillary organ developed at that period upon the thumb, cannot be taken as an indication of the peculiar strength of the sexual instinct. But he would probably admit that battles of gallantry among animals at other times peaceably disposed, or a long series of preliminary *agaceries*, such as Rusconi has described in the salamander, are unquestionable indications of the force of this instinct; and yet among most of the animals which exhibit them, the cerebellum is relatively smaller than in fishes which do not copulate at all, and in which the emission of the semen seems to be an act performed under the same conditions as the contemporaneous extrusion of the ova. The sexual passion appears to be particularly strong amongst serpents; of which, if surprised during the coitus, the male defends the female, exposing himself to every kind of peril for her safety, and submitting to mutilation rather than desert her. The iguana and some other lizards display an equal attachment; and the affection manifests itself not merely during the time of coition, but subsequently; yet its cerebellum is relatively much smaller than that of the crocodile, in which the attention of the male to the female, after copulation, seems limited to helping her on her legs again—her position during the act having been supine. There is nothing in the known manifestations of the sexual passion in this class, that can at all account for the marked superiority in the relative size of the cerebellum of the turtles and crocodiles over that of the frogs and other batrachia.

Now, if we turn to the locomotive actions of this class, we think that a much closer correspondence is apparent. In the first place, the generally small size of the organ accords well with the very limited nature of the movements, and especially with the fact, that these movements are for the most part of a reflex character. The numberless experiments which have been made upon frogs, serpents, &c., fully prove that, not only the ordinary motions of progression, but almost all the actions performed by these animals—even such as seem the most *adaptive* in their character—are of a simply reflex nature. We should fairly expect, then, to find the cerebellum at its minimum of development, especially as, in consequence of the firmness of the support upon which the limbs and body rest in the terrestrial species, there is no need of any exertion to balance the body. The aquatic and semi-aquatic crocodiles, alligators, turtles, and mud-tortoises, however, need a much greater power of combining and regulating the movements of their limbs to sustain, balance, and propel their bodies in the fluid medium, in which, though so helpless on land, they move with great facility; and, accordingly, we find that they have the largest cerebella of the entire class. It may be objected, that there are newts and serpents which are also aquatic, and that these, nevertheless, are not distinguished by a cerebellum of larger size than that of their congeners. But to this it may be replied, that *their* propulsion is not so much effected by the combined and regulated movements of several members, as, like that of ordinary fishes, by the simple undulations of the vertebral column. The inference we have drawn from the comparatively high development of the cerebellum in the turtles, is remarkably confirmed by the fact, that this superiority is confined to the aquatic species: the land tortoise, which can have little need of any co-ordinating power for its simple and stable method of progression, presenting little or no advance, as regards the development of the cerebellum, upon the average of the class.

The cerebellum makes a great advance in development in the class of birds, being not merely of much larger size, but being also marked with numerous transverse lamellæ, which indicate an extension of the surface of gray matter, and being also furnished with lateral lobes, which are the first indications of the hemispheres of the cerebellum. There is not by any means the same difference in its relative size in the several members of this group, as exists in the two preceding classes; and the inferences to be derived from its study, therefore, are less decided than those with which we seem to be furnished by the comparative investigation of its development in fishes and reptiles. Such indications as are afforded, however, by no means favour the idea that this organ is exclusively connected with the sexual passion; for we find that the polygamous fowls, with whose extensive gallantries we are all familiar, have relatively a much smaller cerebellum than the falcons and swallows, which bestow their sexual feelings on a single female. Among the monogamous birds it is very curious to see how constantly, when they have once paired, the male restricts his amorous attentions to his mate, never going astray after any other female, and how uniformly the female rejects the addresses of any other wooer, affording in this respect, as Buffon long since remarked, an excellent pattern to husbands and wives of more elevated rank in the zoological series. Yet this constancy cannot result from the relatively small size of the cerebellum, for the birds which exhibit it have the largest cerebella of the entire class, whilst the licentious chanticleer has among the smallest. Nor can it be said that birds are on the whole less amorous than mammals, in conformity with the inferior development of their cerebellum; for there are no animals in which the size and functional activity of the sexual organs undergo such a marked increase at the period of propagation as they do in birds. On the other hand, the degree of difference which exists in the relative development of the cerebellum, in the several tribes of this class, seems to accord well with the relative amount of necessity which exists for the co-ordination of different muscular actions in the various movements they execute. Thus, the birds which live chiefly upon the ground usually have smaller cerebella than those which spend much of their time on the wing; and, amongst the latter, those which pursue a living prey, and which have need of unusual power of directing and balancing their movements—such as the falcon, kite, and swallow—have a cerebellum exceeding in size the average of the class. The birds of the parrot tribe, which are distinguished by the conformation of their feet, and for the use they make of those members in prehension, also have a very large cerebellum—probably the largest in the entire class—although we are not aware that these birds are remarkable for their amative propensities.

Lastly, in the mammalia we may trace a gradual increase in the development of the cerebellum, from a grade scarcely or not at all above that which it presents in birds, to the large size and remarkable complexity which it possesses in man. This increased development is to be noticed chiefly in the lateral portions or hemispheres, which, instead of being mere appendages to the central portion, gradually come to constitute the chief bulk of the mass. The organ presents its lowest development in the rodentia and marsupialia, many of which are remarkable for their amative propensities, and for the persistence of these, under favorable circumstances, through the whole year. It is somewhat larger and more complex in structure in the ordinary hoofed quadrupeds; and its size and complexity present a further increase in the carnivora. But it is as we ascend through the quadrumanous series, from the four-*footed* rather than four-*handed* lemurs, through the baboons, the monkeys, and the semi-erect apes, up to man, that we observe the most remarkable increase. Now it cannot be affirmed, that there is any corresponding increase in the sexual propensity; for we should be sorry to believe that the well-known lasciviousness of the monkey tribes is surpassed by that of the human race, in a degree at all proportional to the relative size of the cerebellum, which is at least six times larger in the latter than in the former. But there is, on the other hand, as we ascend this series, just such a rapid increase in the number and variety of movements, which the limbs are capable of performing; with an increasing power of balancing the body upon the posterior extremities, which produces an augmented freedom of the anterior; whilst, at the same time, the development of the thumb gives a vast increase to the variety of purposes to which the hands may be applied. None but those who have studied the subject can be aware, what an extraordinary co-ordination of muscular movements is required, for the most common acts of human progression. The erect figure, the small size of the feet, the length of the stride, and a variety of other circumstances, interpose a set of mechanical difficulties, which no human skill has yet been able to overcome. We have seen automata of various kinds,—a writing and drawing automaton, a flute-playing automaton, with divers others,—and more recently a speaking automaton; but we believe that no walking automaton was ever constructed, though the attempt has been often made. In the most remarkable collection we have seen, which included singing birds and crawling spiders,—the latter so well imitated as almost to send some of the ladies present into hysterics,—we remember to have seen one of these attempts; which, although the labour of many years had been bestowed upon it, was nothing more than a figure standing upon two legs, and advancing upon a table by the cautious *sliding* of the feet one before the other, the foot being never lifted from the ground, and not being advanced more than its own length at a time. We notice this circumstance, to show how, in this one act alone, simple as it appears, a much greater and more harmonious combination of different muscular actions is required than is involved in the progression of the lower mammalia; and we need hardly point out, we think, how remarkably man surpasses all others, in the power of adapting his muscular contractions to perform any movement which he desires to execute.

On the other hand, there is nothing either in the strength nor the constancy of the sexual propensity in man, which can at all account for the great superiority in the relative size of his cerebellum over that of every other animal. For the means we have proposed for reconciling the phrenological doctrine of the functions of the posterior lobes with the fact of their absence in the lower mammalia, and their peculiarly large size in man, will scarcely hold good here; since the formation of *ideas*, which are essential to the action of the sexual sensations upon the will, and which are associated with feelings of pleasure,—the combination of the two forming a propensity,—can scarcely be anything else than a *cerebral* operation. And we see no reason whatever for placing the seat of the sexual sensations, and of the origination of respondent movements, anywhere else than in the sensory ganglia, which are evidently their instruments in invertebrata.

It is well known that, among other arguments by which Gall upheld his view of the function of the cerebellum, he maintained that the size of this organ is less in castrated animals than in those possessing their sexual organs entire. He does not, however, appear to have made any very exact observations in support of this position, which he principally rested on a comparison of crania. Subsequent observers have not confirmed the statement. Even M. Vimont admits that the result of his experiments was doubtful. Desirous to put the question to the test, M. Leuret caused his former collaborateur, M. Lassaigne, (of whose qualifications as a scientific observer Mr. Noble expresses his ignorance), to weigh the cerebra and cerebella of a sufficient number of stallions, mares, and geldings, and compare the results. A table of these weights, with that of the medulla oblongata, is given by M. Leuret,* for every one of ten stallions, twelve mares, and twenty-one geldings; and the result is that the cerebellum of the gelding is, on the average, *both absolutely and relatively larger* than that either of the stallion or the mare. The manner in which these results are treated by Mr. Noble is, we are constrained to say, rather too characteristic of the general mode in which phrenologists treat evidence that does not suit their purpose. In the first place, not having seen the entire tables, but only the summary of them given in Dr. Carpenter's 'Human Physiology,' he makes objections which a reference to the tables would prove to be unfounded. His especial ground of refusal to admit the validity of these results, is the smallness of the number of observations, and the consequent uncertainty of the averages; but if he will refer to the tables themselves he will see that even the *extremes* are as remarkable for the evidence they afford as are the *means*. Thus, the weight of the cerebellum of the stallion is to that of the cerebrum in the stallion, as from 1 : 7·46 in the *lowest*, to 1 : 6·25 in the *highest*, the *average* being 1 : 7·07. In the mare the proportional weight of the cerebellum is greater, not from the larger absolute size of that organ, (as Mr. Noble would make out M. Leuret to assert, contrary to what he states is the invariable fact), but from the smaller size† of the cerebrum;

* Anat. Comp. du Systême Nerveux, tom. i, p. 427.

† We have discovered an error of calculation in M. Leuret's table, in regard to the weight of the cerebrum in Mare No. 7. The number (deduced from the weight of the entire encephalon) ought to be 436, instead of 336, and the proportion 6·60, instead of 5·09.

it varies from 1 : 6·93 in the *lowest,* to 1 : 6·08 in the *highest,* the *average* being 1 : 6·59. In the gelding the proportion varies from 1 : 6·44 in the *lowest,* (excluding one case in which the cerebrum was so enormously developed as to reduce the proportional weight of the cerebellum to 1 : 7·44, although the latter organ surpassed, in *absolute* weight, any other which is here recorded), to 1 : 5·16 in the *highest,* the *average* being 1 : 5·97. We cannot but think that any unprejudiced person, on comparing these results, must admit that they are sufficient, at any rate, to countervail the vague statements of Gall, even though they may not be sufficiently numerous to establish an exact average of the relation. Phrenologists have too often shown a great horror of *precise* comparisons; but we submit that here is a case in which there can be no serious mistake, the cerebellum and cerebrum being so completely isolated by Nature that no accident or partiality on the part of the experimenter can affect the results in any considerable degree. We are surprised that a man of Mr. Noble's general candour and benevolence of disposition should have descended to the unworthy but easy method of invalidating the results of experiments which do not accord with his hypothesis, by imputing intentional deceit to M. Leuret, as being a known opponent of phrenology. It will be quite time, we submit, to do this, when Mr. Noble, or any other phrenologist, shall have brought before the world a more extensive series of observations made on the same plan, by which M. Leuret's results shall be overthrown. And even then it will be, of course, for the world to judge from their relative estimate of the two parties, whether the system-makers or the objectors are most likely to be in error. The results of M. Leuret's inquiries have now been before the public for some years; but as we have not heard that they have been thus invalidated, we cannot but admit them as affording important negative evidence with regard to the phrenological view of the functions of the cerebellum.

We have left ourselves no space to touch upon the bearings of pathological observation, with regard to the two views of the functions of the cerebellum. This, however, we the less regret; since the very small number of well-observed cases at present recorded, does not afford any decided evidence either way; it being quite possible, however, to put this evidence in a form that is very favorable to either side, by neglecting all that bears the other way. This Mr. Noble has done, in favour of the phrenological view; recording a small number of coincidences between injuries and diseases of the cerebellum, and disorder of the sexual organs (among others quoting a case, in which the injury was a *flesh wound* in the neck, and in which there was no proof whatever of the cerebellum being affected), but passing over with very imperfect notice the cases in which there was no such coincidence, or in which the phenomena tended the other way. He gives in detail, however, two cases recorded by Dr. Cowan, which seem to have had some influence with that gentleman,—a professed phrenologist, be it observed, but at the same time a man of too philosophical a mind to be a blind partisan of the system—in leading him to view with more favour the doctrines of Flourens. This forfeiture of allegiance to Gall, on the part of one of his followers, very amusingly excites Mr. Noble's surprise; his conviction of the truth of the system being such, that he cannot understand how any one who has once embraced it can relinquish one tittle of its dogmata. And he devotes some space to the analysis of the details of Dr. Cowan's cases, with the view of bringing them into harmony with Gall's doctrine; in this, however, he seems to us to display more ingenuity than philosophy.

On the whole, then, we are disposed to infer, from the remarkable concurrence between the results of experiment and the data supplied by comparative anatomy, that one function of the cerebellum is to regulate and combine the muscular movements required to perform a large number of our voluntary actions. And if the evidence, furnished by observation of the human species only, should prove to be such, as to establish a constant relation between the development of this organ in him, and the strength of his sexual propensity, (a position we by no means dispute, our arguments being only directed against the insufficiency of the evidence on which it rests at present) we must regard this as a *superadded* function, not as the fundamental purpose of the organ.

We conclude, as we commenced, by disclaiming any hostility whatever to the phrenological system in the abstract; and by freely admitting the *general* coincidence between the indications of human character, which are afforded by cranioscopical examination, and those derived from a direct acquaintance. But we consider that, in building up their system, the followers of Gall have been too disregardful of evidence supplied from other sources than observation of man; and that they have been misled, as to the fundamental question of the connexion of the cerebrum with the purely instinctive actions, by their inattention to comparative anatomy, which proves that the cerebrum *cannot* be the instrument of those actions; and have glossed over the important objection which the non-development of the posterior lobes in the lower mammalia and in all the oviparous vertebrata interposes to the location of the animal propensities in them. So far, however, from availing ourselves of these errors, as conclusive arguments against the whole system, we have endeavoured, by a new analysis of the propensities and emotions, to show that the facts supplied by comparative anatomy may be brought into conformity with the physiology of Gall; and that the phrenological system may be planted upon a much more secure and extended basis than it has yet possessed; a new and more exact series of observations, however, being required to build it up with anything like firmness and consistency. We cannot regard the question of the functions of the cerebellum as at all fundamental in its character; and can easily understand how a candid phrenologist like Dr. Cowan may, on this point, embrace the views held (we believe) by all the leading physiologists of the day.

Finally, we commend our review of the subject to the candid consideration of those who think with us that the determination of the *general* functions of the *encephalon* is the *first* question for the physiologist; that the determination of the share of these performed by the *cerebrum*, to be effected by attention to comparative anatomy and by experiment, is the *second;* and that the determination of the *special* functions of *different parts of the cerebrum,* to be effected (for the reasons we have stated) by the comparison of the varieties of cerebral (not cranial) conformation in man, with some assistance from that of the lower animals, is the *third.* Upon those who blindly uphold the method of Gall, in disregard of all the improvements which have taken place in our knowledge of neurology since his time, and who regard the present phrenological system as so perfect as to be incapable of improvement, we certainly despair of making any impression. We would not be supposed to assert our conviction that our own views as now expressed, are so complete as to be incapable of further improvement. Considering as we do that the whole science of encephalic phrenology is in its infancy,—that a large amount of information as to the fundamental data on which it must be built up is yet wanting,—that the progress of comparative anatomy, of embryology, and of microscopy, may make many additions to our knowledge of the connexions of different parts of the nervous centres which may tend to modify previously-received doctrines,—and that, on the other hand, an entirely new system of psychological observation must be carried out, in order to bring psychology and physiology into their proper relation;—it would be absurd in us to attempt to lay down dogmatic conclusions, by which to stand or fall. We would be understood as attempting nothing in this article, but to test the relative validity of two rival *methods* of philosophizing on this subject. Our conviction of the uniformity of Nature is such, that we are thoroughly persuaded that there can be no real contradiction between her various indications, when these are properly brought together and compared; and our attempt has been to point out the application of the method, which has been elsewhere pursued with complete success, to neurological investigation. We deem it particularly incumbent upon us to point out that even if our views should be proved to be erroneous as to a few minor points,—such, for example, as the offices of the thalami and corpora striata,—our main argument is not affected. The points for which we contend are simply these:—the independent character of the sensory ganglia as the instruments of sensation and of consensual actions,—the superadded character of the cerebrum, as the organ by whose instrumentality ideas are formed and reasoning processes are carried on,—and the mixed character of the emotions and propensities, as compounded of *ideas* and the *simple feelings* of pleasure and pain. On this last point, we venture to think that we have made a real advance in psychology, which will prove to be important; and we happen to know that several intelligent psychologists are well prepared to receive it, as fixing and defining views which had been previously floating in their own minds. It seems, indeed, to have been glimpsed at by the late Mr. James Mill, in his valuable 'Analysis of the Human Mind;' his deficiency consisting in connecting the feeling too much with the sensation, rather than with the intellectual idea. We should be doing injustice to that very pains-taking anatomist, Mr. Swan, were we not to state that, on referring to his general summary of his views of the offices of the nervous centres, we find a very near coincidence with the leading features of our own doctrines regarding the relative offices of the sensory ganglia and the cerebrum;—doctrines, indeed, to which it would be easy to point out approaches in the writings of many previous physiologists, to whose authority we might refer in support of our own.

